# Systems Biology Analysis of the Radiation-Attenuated Schistosome Vaccine Reveals a Role for Growth Factors in Protection and Hemostasis Inhibition in Parasite Survival

**DOI:** 10.3389/fimmu.2021.624191

**Published:** 2021-03-11

**Authors:** Leonardo Paiva Farias, Juliana Vitoriano-Souza, Lucas Esteves Cardozo, Leonardo Dos Reis Gama, Youvika Singh, Patrícia Aoki Miyasato, Giulliana Tessarin Almeida, Dunia Rodriguez, Mayra Mara Ferrari Barbosa, Rafaela Sachetto Fernandes, Tereza Cristina Barbosa, Almiro Pires da Silva Neto, Eliana Nakano, Paulo Lee Ho, Sergio Verjovski-Almeida, Helder Imoto Nakaya, Robert Alan Wilson, Luciana Cezar de Cerqueira Leite

**Affiliations:** ^1^ Laboratorio de Desenvolvimento de Vacinas, Instituto Butantan, São Paulo, Brazil; ^2^ Laboratório de Inflamação e Biomarcadores, Instituto Gonçalo Moniz, Fundação Oswaldo Cruz, Salvador, Brazil; ^3^ Faculdade de Ciências Farmacêuticas, Universidade de São Paulo, São Paulo, Brazil; ^4^ Laboratorio de Parasitologia, Instituto Butantan, São Paulo, Brazil; ^5^ Instituto de Química, Universidade de São Paulo, São Paulo, Brazil; ^6^ Programa de Pós-Graduação Interunidades em Biotecnologia—USP-Butantan-IPT, São Paulo, Brazil; ^7^ Centro BioIndustrial, Instituto Butantan, São Paulo, Brazil; ^8^ York Biomedical Research Institute, University of York, York, United Kingdom

**Keywords:** *Schistosoma mansoni*, radiation-attenuated vaccine, systems biology, immune response, protection mechanism, mouse model

## Abstract

In spite of several decades of research, an effective vaccine against schistosomiasis remains elusive. The radiation-attenuated (RA) cercarial vaccine is still the best model eliciting high protection levels, although the immune mechanisms have not yet been fully characterized. In order to identify genes and pathways underlying protection we investigated patterns of gene expression in PBMC and skin draining Lymph Nodes (LN) from mice using two exposure comparisons: vaccination with 500 attenuated cercariae versus infection with 500 normal cercariae; one versus three doses. Vaccinated mice were challenged with 120 normal parasites. Integration of PBMC and LN data from the infected group revealed early up-regulation of pathways associated with Th2 skewing and polarization of IgG antibody profiles. Additionally, hemostasis pathways were downregulated in infected mice, correlating with platelet reduction, potentially a mechanism to assist parasite migration through capillary beds. Conversely, up regulation of such mechanisms after vaccination may explain parasite blockade in the lungs. In contrast, a single exposure to attenuated parasites revealed early establishment of a Th1 bias (signaling of IL-1, IFN-γ; and *Leishmania* infection). Genes encoding chemokines and their receptors were more prominent in vaccinated mice, indicating an enhanced capacity for inflammation, potentially augmenting the inhibition of intravascular migration. Increasing the vaccinations from one to three did not dramatically elevate protection, but there was a clear shift towards antibody-mediated effectors. However, elements of the Th1 bias were still evident. Notable features after three vaccinations were markers of cytotoxicity (including IL-6 and NK cells) together with growth factors and their receptors (FGFR/VEGF/EGF) and the apoptosis pathway. Indeed, there is evidence for the development of anergy after three vaccinations, borne out by the limited responses detected in samples after challenge. We infer that persistence of a Th1 response puts a limit on expression of antibody-mediated mechanisms. This feature may explain the failure of multiple doses to drive protection towards sterile immunity. We suggest that the secretions of lung stage parasites would make a novel cohort of antigens for testing in protection experiments.

## Introduction

Schistosomiasis is a debilitating disease caused by flatworms of the genus *Schistosoma*. It represents a serious public health problem for which various control measures are available but fail to prevent re-infection in endemic areas (1). In order for schistosomes to establish in the mammalian host, cercariae must penetrate the skin, transform into schistosomula, and migrate in the bloodstream to the hepatic portal venules of the liver. There they grow into pre-adults, pair and travel up the portal vasculature to their favored sites in the intestinal wall or bladder venous plexus. Even today, diagnostics for schistosomiasis are not very sensitive and cannot detect low-level infections (2). In this context, an effective vaccine with long-term protective effects *via* the activation of the immune system would be the ideal tool to prevent this disease (3). Since the parasite does not replicate in its definitive host, it is evident that even a partial reduction in parasite burden would have an impact on disease morbidity, control and ultimately eradication (4).

In the last decades numerous studies have been conducted in an attempt to develop a vaccine against schistosomiasis, but with the exception of studies with Sm14, Calpain, and TSP-2 antigens, the results were not very encouraging (5). Most viral or microbial vaccines are intended to replicate events after primary exposure that naturally elicit solid immunity. With chronic infections, like schistosomiasis, the vaccinologist starts at a disadvantage in that the existence of a protective immune response is by no means clear or guaranteed (6). Accordingly, there are no obvious human paradigms on which to base a schistosome vaccine, and researchers have focused their strategies on immune responses in animal models (7).

For many pathogens, the most effective vaccines comprise the live infectious agent that has been attenuated to make it non-pathogenic (8, 9). In the case of *Schistosoma*, studies with radiation-attenuated (RA) larvae have demonstrated that a schistosomiasis vaccine is a realistic goal [reviewed in (10)]. Parameters for immunization of mice with ^60^Cobalt-irradiated *Schistosoma mansoni* cercariae were first described by Minard et al. (11) and resulted in protection against a subsequent challenge infection. In the ensuing decades, the *S. mansoni* RA cercarial vaccine has been tested experimentally in different animal models and provided important insights, especially in studies with knockout mice (12, 13). It is clear from numerous studies on the RA vaccine (10) that attenuated parasites must persist in the host for 1 to 2 weeks to elicit effective protection. “The lung is the major site of parasite attrition and elimination for both immunizing and challenge infections in the mouse model” [reviewed by (14)]. Nevertheless, many of the immune events following vaccination with attenuated cercariae are still poorly understood. Therefore, more detailed studies with the *Schistosoma* attenuated vaccine are important to identify pathways that correlate with protective immunity, contributing to research on, and development of human vaccines.

In this context, a systems biology approach can provide an overview of the immune system and its many components by analyzing the coordinate interactions at the molecular level and can help overcome current challenges in vaccine development. Systems vaccinology is an emerging field in the study of immune responses to vaccination, combining the measurement of different parameters, analysis of their interaction network and predictive modeling using bioinformatics tools applied to vaccinology (15–17). This new approach is highly relevant to the search for a vaccine against schistosomiasis and will help the efforts to explain “how and why” RA cercariae generate high protection levels against subsequent challenge, whereas a natural infection does not.

Systems biology has been used to understand the immune response induced by vaccination against yellow fever (18), influenza (19, 20), as well as for malaria (21, 22) and HIV infection (23, 24). Querec and collaborators, through the analysis of genes activated in PBMC using microarrays, have identified molecular signatures able to predict the early events in the immunogenicity of YF-17D, the attenuated yellow fever vaccine (virus) in humans (18). Additionally, Nakaya and collaborators compared the gene networks in humans immunized with two different inactivated (TIV) and attenuated (LAIV) influenza vaccines (20). The results showed the ability of systems biology approach to predict the immunogenicity of these vaccines and revealed new molecular mechanisms involved in immunity. A central aspect of these studies was the design of time-course blood sampling to assess the PBMC transcriptomes.

To date, there have been few studies analyzing changes in host gene expression to evaluate the efficacy of vaccines against schistosomiasis. Tian et al. (25) investigated immunological events induced by vaccination of pigs with ultraviolet (UV) attenuated cercariae of *S. japonicum* and protection after experimental challenge. More recently, Rojo et al. (26) employed RNA sequencing to identify gene signatures that may predict calpain vaccine efficacy in mouse and baboon models. In the present study, we have explored the murine RA vaccine model by analyzing the early sdLN response and PBMC transcriptional profiles over a time course after vaccination and challenge in relation to a series of parameters: hematology, antibody profile, blood immunophenotypic profile, and parasite burden. Our aim was to seek answers to two key questions concerning the *modus operandi* of the RA vaccine at the level of host immune gene expression about why: 1) attenuated larvae elicit a protective response while normal larvae do not; 2) multiple exposures to the RA vaccine change the balance from a Th1 to a Th1-Th2 profile but nevertheless fail to drive protection towards sterile immunity.

## Materials and Methods

This study was carried out using C57BL/6 female mice aged 6–8 weeks, and all experiments were conducted in strict accordance with good practices as defined by the Committee for the Ethical Use of Animals in Experimentation of the Butantan Institute (São Paulo, Brazil) under license 1030/13. *S. mansoni* (BH strain) was maintained in a laboratory by routine passage through hamsters and *Biomphalaria glabrata* snails as previously described (27). Parasites used for experimental vaccination were attenuated by exposure to gamma ray irradiation (20krad, 200Gy) using a Gamma cell 220 ^60^Co source (Atomic Energy Agency of Canada Ltd.) at room temperature in the presence of O_2_ located at the radiation technology center (CTR-IPEN).

### Vaccination Trials and Challenge Procedures

**Figure 1:**
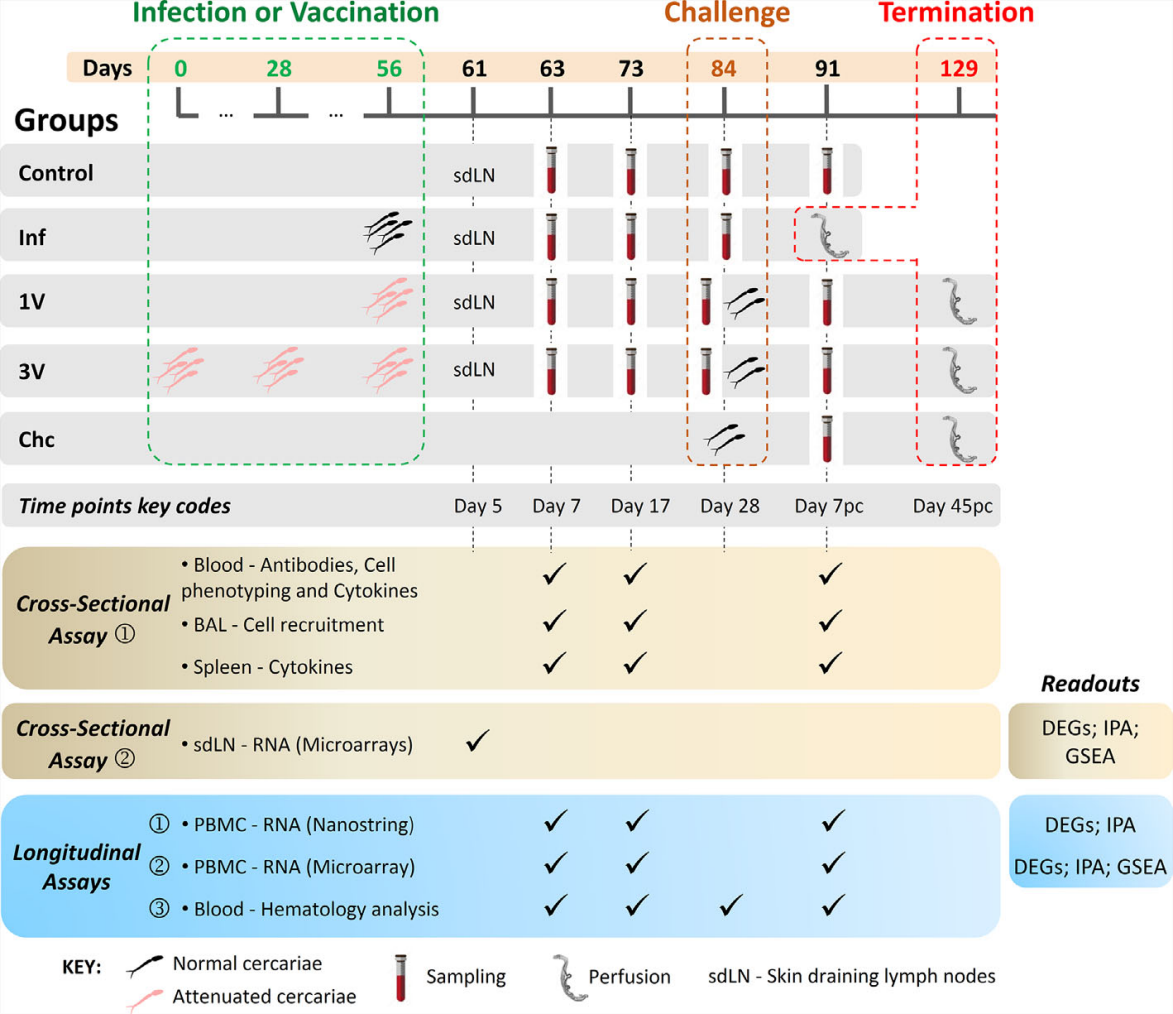
Experimental design of immunization/infection procedures and collected material at the indicated time points. C57BL/6 mice were disposed in five experimental groups: Control (C) non-treated animals; (1V) animals vaccinated with one dose of 500 attenuated cercariae; (3V) animals vaccinated with three doses of 500 attenuated cercariae; (Inf) animals infected with 500 normal cercariae in parallel with the immunizations and perfused at Day 91; animals only challenged with 120 normal cercariae (Chc). Vaccinated and Chc groups were challenged with 120 normal cercariae at 84 days. The vaccination/infection and the challenge occurred at 4-week intervals. Blood, BAL, and spleen analyses were performed at Day 7 and 17 after vaccination/infection and Day 7 post-challenge. Protection was assessed at the end of the experiment by portal perfusion, 45 days post-challenge. Lower panels display the cross-sectional and longitudinal assays performed, and readouts assessed: Cross-sectional assay➀, in which 72 mice were divided in five experimental groups (C, Inf, 1V, 3V and Chc) and six mice per group were euthanized at the different time points (Day 7, 17 and 7 pc) for immune response evaluation in the blood (antibodies; cell phenotyping; cell recruitment to lungs by bronchoalveolar lavage (BAL); splenic and blood cytokines). Cross-sectional assay➁, in which 40 mice were divided in four experimental groups (C, Inf, 1V, and 3V) and 10 mice per group were euthanized at Day 5 after vaccination/infection for transcriptome analysis of the skin draining lymph nodes (sdLN); Longitudinal assay➀ and ➁, for each assay 72 mice were divided in five experimental groups (C, Inf, 1V, 3V and Chc; 12-15 mice per group) and the PBMC was isolated for immune transcriptome profiling by NanoString and for global transcriptome analysis by microarray, respectively; Longitudinal assay➂, in which 50 mice were divided in five experimental groups (C, Inf, 1V, 3V and Chc; 10 mice per group) for hematological evaluation and sera separation for epitope mapping with peptide arrays (28). For all longitudinal assays, worm burden was evaluated upon the termination of experimentation.

Cross-sectional and longitudinal assays were performed to investigate differences among aspects of the immune response induced after vaccination, infection and challenge. C57BL/6 mice were distributed into five groups: control (C), non-treated animals; vaccinated once (1V) with 500 attenuated cercariae and challenged with 120 normal cercariae; vaccinated three times (3V) with 500 attenuated cercariae (4-week intervals) and challenged with 120 normal cercariae; only challenged with 120 normal cercariae at 84 days (Chc); infected with 500 normal cercariae (Inf) in parallel with the 1V group. The Inf group was perfused 35 days after infection (91 days after the beginning of the experiment; **Figure 1**). The inclusion of the Inf group was designed to detect quantitative and qualitative differences in the immune response induced by identical numbers of normal and attenuated cercariae. Mice were anaesthetized using 80 mg/kg of ketamine (Dopalen) and 10 mg/kg of xylazine (Anasedan; both from Ceva Santé Animale, Paulínia, SP) and infected or vaccinated by percutaneous exposure of shaved abdominal skin for 30 min (29) with 500 normal or 500 attenuated cercariae at the time points indicated in **Figure 1**. Four weeks after the final vaccination, the 1V and 3V mice were challenged with 120 normal cercariae by the tail method (30). Forty-five days after challenge, all animals were euthanized with a lethal dose of anesthetic (ketamine and xylazine), and perfusion fluid (saline solution plus 500 units/L of heparin) was pumped into the dorsal aorta allowing for the collection of perfused worms from the ruptured hepatic portal vein. The intestinal mesenteric vessels were examined for residual trapped worms, after which all collected worms were counted using a stereomicroscope.

For the Inf group sampling beyond 5 weeks was not possible because of the lethality of this parasite burden.

### Experimental Design

The choice of sampling time points was based mainly on the work of Ratcliffe and Wilson (31), who described the peak of the systemic response (response of parasite-specific T-lymphocytes) as occurring 17 days after vaccination, and the peak of recall reactivity after a challenge as occurring seven days after secondary exposure. Cross-sectional and longitudinal assays were performed as follows (**Figure 1**):

- Cross-sectional assay➀, in which 72 mice were divided in five experimental groups (C, Inf, 1V, 3V and Chc), and six mice per group were euthanized at Days 7 and 17 after infection or vaccination, and at Day 7 post-challenge (Day 7 pc) for immunological evaluation (antibodies; blood cell phenotyping; cell recruitment to lungs by bronchoalveolar lavage (BAL); splenic and blood cytokines);
- Cross-sectional assay➁, in which 40 mice were divided in four experimental groups (C, Inf, 1V, and 3V) and 10 mice per group were euthanized at Day 5 after vaccination/infection for transcriptome analysis of the inguinal and axillary lymph nodes draining the skin (sdLN);
- Three independent longitudinal assays, in which blood was drawn from the same groups of mice in a time-course manner at Days 7 and 17, and Day 7 pc as follows:
- Longitudinal assay➀, in which 72 mice were divided in five experimental groups (C, Inf, 1V, 3V and Chc; 12–15 mice per group) and PBMC was isolated for immune transcriptome profiling by NanoString;
- Longitudinal assay➁, in which 72 mice were divided in five experimental groups (C, Inf, 1V, 3V and Chc; 12–15 mice per group) and PBMC was isolated for global transcriptome analysis by microarray. In this experimental block, data from ten additional animals from a pilot standardization assay were added to the control group with a total of 22 animals;
- Longitudinal assay➂, in which 50 mice were divided in five experimental groups (C, Inf, 1V, 3V and Chc; 10 mice per group) for hematological evaluation and sera separation for epitope mapping with peptide arrays (28). For all longitudinal assays, worm burden was evaluated upon the termination of experimentation (**Figure 1** and **Supplementary Figure 2**).

### Assessment of Vaccine-Induced Protection: Adult Worm and Egg Recovery

For the three independent longitudinal assays, the protection level (%) in vaccinated mice was calculated after perfusion by comparing the number of worms recovered from each vaccinated group with the control group, as previously described (32). To evaluate the liver-trapped egg burden, a piece of the liver from each mouse was removed, weighed, digested and homogenized for 1 h at 37 °C in 5 ml of 10% KOH, the number of eggs per gram of liver was compared to the control group. Worm fecundity was calculated by dividing the number of eggs/gram of liver tissue by the number of female worms collected. Worm size was assessed by measuring the area of each worm using ImageJ software (http://rsb.info.nih.gov/ij) following the acquisition of images using a camera (DINO-Eye, AM423X, Anmo Electronics Corporation, Taiwan) coupled to a light microscope (NIKON – Eclipse E200, Japan).

### Bronchoalveolar Lavage (BAL)

At Days 7, 17 and Day 7 pc, six mice per group per time point derived from cross-sectional assay➀ were euthanized by an i.p. injection of ketamine and xylazine and several immunological parameters were assessed. After euthanasia, the trachea was cannulated and each animal’s lungs were washed twice with 0.5 and 1.0 ml of cold PBS (phosphate-buffered saline, pH 7.4), and the recovered material collected in microtubes. After total cell counting, cytospin preparations of BAL cells were stained with Instant-Prov (Newprov), and differential cell counting was performed on 200 cells according to respective morphological and staining characteristics.

### Blood Cell Immunophenotyping by Flow Cytometry

Blood cell phenotyping was performed by flow cytometry using cells derived from six mice per group per time point (cross-sectional assay➀). Cells were stained with monoclonal antibodies against CD3 (APC-Cy7™ Rat anti-mouse CD3 Molecular Complex, clone17 A2, BD Pharmingen™), CD4 (PE-Cy™ 5 Rat anti-Mouse CD4, clone H129.19, BD Pharmingen™), CD8 (PE™ Rat anti-Mouse CD8a, clone 53–6.7, BD Pharmingen™) and the early T cell activation marker CD69 (FITC Hamster anti-Mouse CD69, clone H1.2F3, BD Pharmingen™). All immunophenotyping and gate strategy was performed as previously described (33) and exemplified in **Supplementary Figure 3A**. Flow cytometry measurements were performed on a FACSCanto II ^®^ instrument (Becton Dickinson, Moutain View, CA) with 15,000 events acquired for each preparation. FlowJo Software (Flow Cytometry Analysis Software 7.6, Tree Star, Inc., Ashland, OR) was used for data analysis.

### IgG and IgE Antibody Levels

Six mice per group per time point derived from cross-sectional assay➀ were bled from the retro orbital plexus and sera were collected for ELISA assays to assess anti-schistosome IgG, IgG1, IgG2c and total IgE antibody levels. For the quantification of IgG, IgG1 and IgG2c, 96-well plates (Maxisorp 96-well microtiter, Nunc) were coated with 1 µg/ml of 7-day-old schistosomule total protein extract (34) in carbonate-bicarbonate buffer (pH 9.6) as described previously (27). Total IgE ELISA was carried out using a Mouse IgE ELISA kit (BD OptEIA TM, BD Biosciences, USA) using sample dilutions (1:40) in accordance with manufacturer recommendations. IgG, IgG1, and IgG2c antibody levels were calculated using a standard curve and horseradish peroxidase (HRP)-conjugated antibody anti-mouse IgG, IgG1, or IgG2c (Southern Biotechnology). IgE antibody levels were expressed as OD (optical density) with readings performed at 492 nm in an ELISA plate reader (Epoch Biotek, Biotek Instruments Inc., USA). The correlation analysis of antibody levels at Day 28 and parasitic load, as well as the ratio analysis of IgG1/IgG2c were performed with data collected from longitudinal assay➁ with 15 mice per group.

### Cytokine Measurements in Blood and Spleen Cultures

Cytokine assays were assessed in whole blood (without stimulus), from six mice per group per time point derived from cross-sectional assay➀, as previously described (35). Spleens were removed aseptically and processed as previously described (36). Cell viability counts were performed after staining with Trypan blue. Cells (1x10^6^ per well) were plated on 24-well plates and stimulated with schistosomula extract (15 µg/ml) or concanavalin A (5 µg/ml, Sigma) as a positive control for cell reactivity, or saline as a negative control. After 72 h, culture supernatants were collected for cytokine assay using a Mouse Th1/Th2/Th17 CBA kit (BD™ Cytometric Bead Array). All procedures were conducted according to the manufacturer’s recommendations. Data was plotted by subtracting the baseline levels of cytokine production (no stimulus) for each group, and then displayed as a heatmap (z-score).

### Hematological Evaluation

Fifty microliters of peripheral blood were collected from the retro-orbital complex of 10 mice per group derived from longitudinal assay➂ and transferred to tubes containing 10% EDTA (Sigma Chemical Co) as an anticoagulant. An Auto Hematology Analyzer apparatus (Mindray BC-2800Vet, Hamburg, Germany) was used to evaluate parameters related to white blood cells (total leukocytes, lymphocytes, monocytes and polymorphonuclear cells) and platelets. Data was calculated as the fold change relative to the control group and then displayed as a heatmap.

### Immune Transcriptional Profiling of PBMCs by NanoString Analysis

To obtain the immune transcriptional profiles of PBMCs after infection, immunization, and challenge, the NanoString nCounter Gene Expression Platform (NanoString Technologies, Inc.) was employed. The Nanostring system utilizes a digital barcode technology for the multiplexed measurement of analytes and offers high levels of precision and sensitivity (< 1 copy per cell) (37). The nCounter^®^ Mouse Immunology panel used herein covers ~546 general immunology genes. For this experimental block, nine mice per group derived from the longitudinal assay➀ were evaluated (**Supplementary Table 1**). Blood collection, PBMC separation and RNA extraction were performed as described in **Supplementary Methods**. These assays were performed at a NanoString Technologies facility (Seattle, WA, USA) using 200 ng of total RNA in accordance with the manufacturer’s recommendations. Three independent biological replicates were assessed per group per time point, in which each individual biological replicate consisted of a pool of PBMCs (containing equal amounts of total RNA) from three mice (**Supplementary Table 1**). In summary, gene expression levels were measured by counting the number of times the color-coded barcode for a given gene was detected (digital counting). Normalization, expression values (fold change calculations) and statistical analyses were performed using nSolver™ Analysis Software (version 2.6).

### Global Gene Expression Profiling of PBMCs and sdLN by Microarray

To obtain a comprehensive overview of the pathways operating under different experimental conditions, we analyzed the PBMC transcriptional profiles obtained with Agilent DNA arrays after vaccination, infection and challenge (SurePrint G3 Mouse GE 8x60K Microarray, Agilent, USA). This DNA array comprises 39,430 Entrez Gene coding transcripts and 16,251 non-coding transcripts from the mouse genome (RefSeq Build 37). For this experimental block, 12–15 mice per group (Inf, 1V, 3V and Chc) and 22 mice for control group (C) derived from the longitudinal assay➁ were evaluated. Blood collection, PBMC separation and RNA extraction were performed as described in **Supplementary Methods**. Data revealed a high correlation between the number of PBMCs counted and the amount of RNA extracted (**Supplementary Figure 1**). Four to five independent biological replicates were assessed per group (Inf, 1V, 3V, and Chc) per time point, and 16 biological replicates from control group (C) were used to generate the baseline of expression for PBMC microarray analysis. All biological replicate consisted of a pool of PBMCs (containing equal amounts of total RNA) from three mice (**Supplementary Table 2**).

We also used the Agilent array to evaluate gene expression profiles in the skin draining lymph nodes at Day 5 after vaccination/infection. For this, 10 mice per group were euthanized and the sdLNs (axillary and inguinal) were collected, fixed overnight in RNAlater solution (Ambion) and then stored in liquid nitrogen until RNA extraction, performed using a RNeasy Micro Kit (QIAGEN) as per manufacturer’s recommendations. Three independent biological replicates were assessed per group for sdLN microarray analysis, in which each biological replicate consisted of a pool of sdLNs from three mice (containing equal amounts of total RNA – RIN > 7) (**Supplementary Table 2**).

To produce Cy3-labeled cRNA, each pool of total RNA was amplified and labeled using an Agilent Low Input Quick Amplification Labeling Kit in strict accordance with manufacturer instructions (**Supplementary Methods**). Data were corrected using a quantile normalization method and transformed into logarithmic (log_2_) values, which allowed for direct comparisons between up-regulated and down-regulated genes.

### Analysis of Expression Data

To identify differentially expressed genes (DEG), a standard pipeline was implemented for probe filtering and expression values obtained from normalized data were consolidated, and then corrected for batch effects. First, the control probes from Agilent were eliminated, followed by the probes that did not map to known genes according to http://biodbnet.abcc.ncifcrf.gov/db/db2db.php. Next, the probes with no significant signal (flag gIsPosAndSignif Agilent) in at least half of the analyzed replicates were removed. Finally, median values were considered for probes replicated within a given array, while in the case of different probes that mapped to the same gene, the greatest mean expression value was considered. These procedures were performed separately with respect to each experimental time point, resulting in a final list of between 17,000 and 18,000 genes. The fold change calculations and differentially expressed gene (DEG) identification, relative to the control group for each condition was performed using the limma R package. A moderate t-test for each gene in each condition was performed, and then the generated p-value was adjusted by false discovery rate (FDR). Due to the small range in gene expression observed for the Volcano plots and Venn diagram analysis we have selected DEGs using a cutoff p ≤ 0.001 (not adjusted by FDR).

To evaluate the genome-wide expression profiles and determine whether *a priori* a defined set of genes showed statistically significant, cumulative changes in gene expression correlated with the different vaccination regimens, Gene Set Enrichment Analysis (GSEA, Broad Institute; https://www.broadinstitute.org/) was performed at each time point, using the normalized values of signal intensity in the microarray of all genes in the samples of one test condition and the control samples. This tool evaluates whether the cumulative changes in gene expression in one tested condition is correlated with a particular previously characterized group of multiple genes (a gene set) defined based on prior biological knowledge. Procedures for GSEA were performed as described in **Supplementary Methods**. Pathway analysis was performed using Ingenuity Pathway Analysis software (IPA) (Qiagen) as described in **Supplementary Methods**.

### Statistical Analysis

Differences in worm and egg burdens, worm size, antibody levels and cell counting were analyzed using a one-way ANOVA followed by Tukey’s multiple post-hoc comparison test. Differences were considered statistically significant when P ≤ 0.05. All graphs, Pearson’s correlation coefficient and statistical analyses were performed using PRISM version 5.02 software (GraphPad, San Diego, CA).

## Results

### Protective Efficacy Induced by Radiation-Attenuated (RA) Vaccination

**Figure 2:**
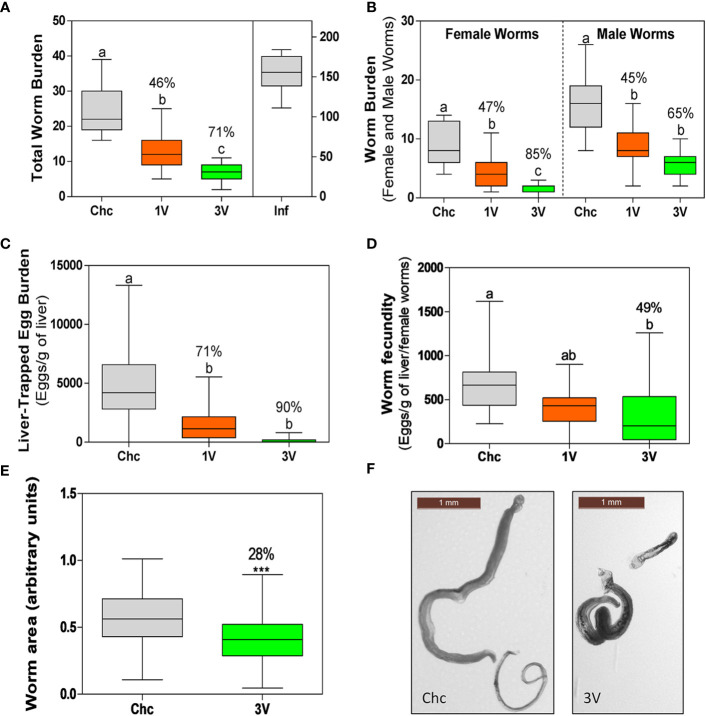
Parasitological parameters of the longitudinal assay➁. **(A)** Total worm burden and protection (%) for one-vaccine dose (1V) and three vaccine doses (3V) in comparison to challenge control (Chc). The Inf group was infected with 500 normal cercariae. **(B)** Worm burden separated by schistosome gender (male and female worms) and protection (%) in comparison to Chc. **(C)** Liver-trapped eggs and **(D)** Egg load per gram of liver per female. The percentage protection is presented for 1V and 3V groups in relation to the challenge control (Chc). **(E)** Worms with impaired development recovered from mice vaccinated with three doses (3V) (***P < 0.001). Area was measured for 177 worms from Chc and 101 worms from 3V, using the ImageJ program. **(F)** Representative images of worms of the Chc and 3V groups. Box plots with different letters represent significant differences and box plots sharing at least one letter represents no significant difference by ANOVA, Tukey (p<0.05); the Inf group was not included in this analysis. Data collected from longitudinal assay➁ (15 mice per experimental group) and representative of two additional longitudinal assays➀ and ➂ (detailed in **Supplementary Figure 2**).

Initially, the protection levels and consistency of the RA vaccine were validated. The animals immunized with a single dose of attenuated cercariae (1V) showed on average of 46% worm burden reduction (47% female, 45% male worms). Protection was higher in the 3V group, representing a 71% worm burden reduction (85% female, 65% male worms). No gender bias reduction was detected, as differences between male and female reduction were proportional to the differences observed in the Chc group (**Figures 2A, B**). Egg count analysis in the liver revealed oviposition reduction, with a 71% decrease in the 1V and 90% in 3V groups, relative to the Chc group (**Figure 2C**). Furthermore, when these data were normalized to the number of females, a 49% reduction of fecundity was observed in the 3V relative to Chc group (**Figure 2D**). Lastly, the body lengths of worms recovered from 3V were 28% shorter than Chc animals under morphometric analysis and several presented a stunted phenotype (**Figures 2E, F**). This immunization and challenge assay was performed two more times and presented very similar results, reinforcing the consistency of the RA cercarial vaccine (**Supplementary Figure 2**).

### Immunization With RA Cercarial Vaccine Induces Immune Responses in the Lungs and Blood

The analysis of bronchoalveolar lavage (BAL) revealed that mice vaccinated with attenuated cercariae (1V and 3V) presented increased immune cell migration in comparison to infected (Inf and Chc) and control animals (**Supplementary Figure 3B**). The 3V recruitment was already elevated by Day 7, and in both immunized groups at Day 17, which was maintained at Day 7 pc (**Supplementary Figure 3B**). There was a predominance of macrophages and lymphocytes, with increased neutrophils at Day 7 and increased eosinophils at Day 17 (**Supplementary Figure 4**). Notably, the infection and challenge protocols involving two different doses of normal cercariae (Inf or Chc) did not elicit significantly altered BAL profiles.

We anticipated that parasite migration through the skin and bloodstream would promote alterations (e.g. tissue damage, inflammation and the healing process) that could be detected systemically by blood analysis, without the need for *in vitro* stimulation. Phenotypic blood analysis revealed the higher levels of activated T cells (CD4^+^ and CD8^+^) on Day 17 in the 3V than 1V group, which were in turn higher than Inf and Control groups (**Supplementary Figure 3C**). In general, the impact of vaccination was to generate higher total leucocyte and platelet levels in the blood (3V > 1V > Inf) as revealed by the hematological evaluation (**Supplementary Figure 3D**).

**Figure 3:**
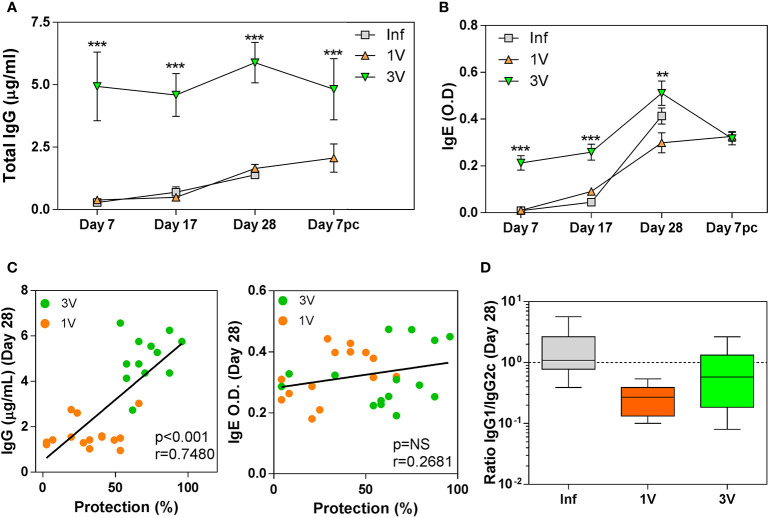
Antibody levels in serum of vaccinated/infected mice: **(A)** Total schistosome-specific IgG antibody and **(B)** IgE in 1V, 3V and Inf at Days 7, 17 and 28 plus Day 7 pc; asterisks indicates significant differences in relation to the Infected group (Inf), ANOVA followed by Tukey’s post-hoc test (**p < 0.01, ***p < 0.001). **(C)** Correlation analysis of total schistosome-specific IgG and total IgE at Day 28 with worm burden at experiment termination in the immunized groups **(D)** Box-plot and whisker graph representing the lower and upper quartiles, median and minimum- maximum values for IgG1/IgG2c ratios in the serum of immunized mice at Day 28. Data derived from cross-Sectional➀ for **(A, B)** (six mice per group per time point) and derived from longitudinal assay➁ (15 mice per experimental group) for **(C, D)**.

In the interpretation of antibody responses, it is important to note that time point 0 is in relation to 1V/Inf, so 3V animals were sensitized by two previous exposures to 500 RA cercariae (**Figure 1**). Thus, high levels of total anti-*Schistosoma* IgG were observed in the 3V group at all time points analyzed, whereas levels increased gradually in parallel in the 1V and Inf groups to Day 28, and again after challenge of the 1V animals (**Figure 3A**). The pattern for the IgE isotype followed a similar trajectory with a maximum reached at Day 28, but the differences between groups were not statistically significant (**Figure 3B**). Also, at Day 28, a significant negative correlation between antibody levels and final worm burden was observed for IgG, but not for IgE (**Figure 3C**). Our analysis of the IgG1/IgG2c subclass ratio at Day 28 indicated an immune response biased towards a Th1 profile in the 1V group, whereas the Inf group was more polarized towards a Th2 profile, and the 3V group was intermediate between the two (**Figure 3D**).

The cytokine production by cultured splenocytes after *in vitro* stimulation revealed an early and intense immune response at Day 7, which was maintained until Day 17, with little distinction between the 1V, 3V and Inf groups; this was characterized by the production of both proinflammatory, Th1 and regulatory cytokines (**Supplementary Figure 5A**). The analysis of plasma cytokines revealed that the previously primed 3V group already had the highest circulating levels of inflammatory (IL-6, TNF-α), Th1 (IFN-γ), Th2 (IL-4) and regulatory (IL-10) cytokines at Day 7; this pattern was maintained at Day 17, and Day 7 post-challenge (**Supplementary Figure 5B**). The 1V group also showed increases in IL-4, IFN-γ and IL-6 at Day 17 (**Supplementary Figure 5B**).

### Infection and Immunization Events Induce Different Gene Expression Profiles in PBMCs

**Figure 4:**
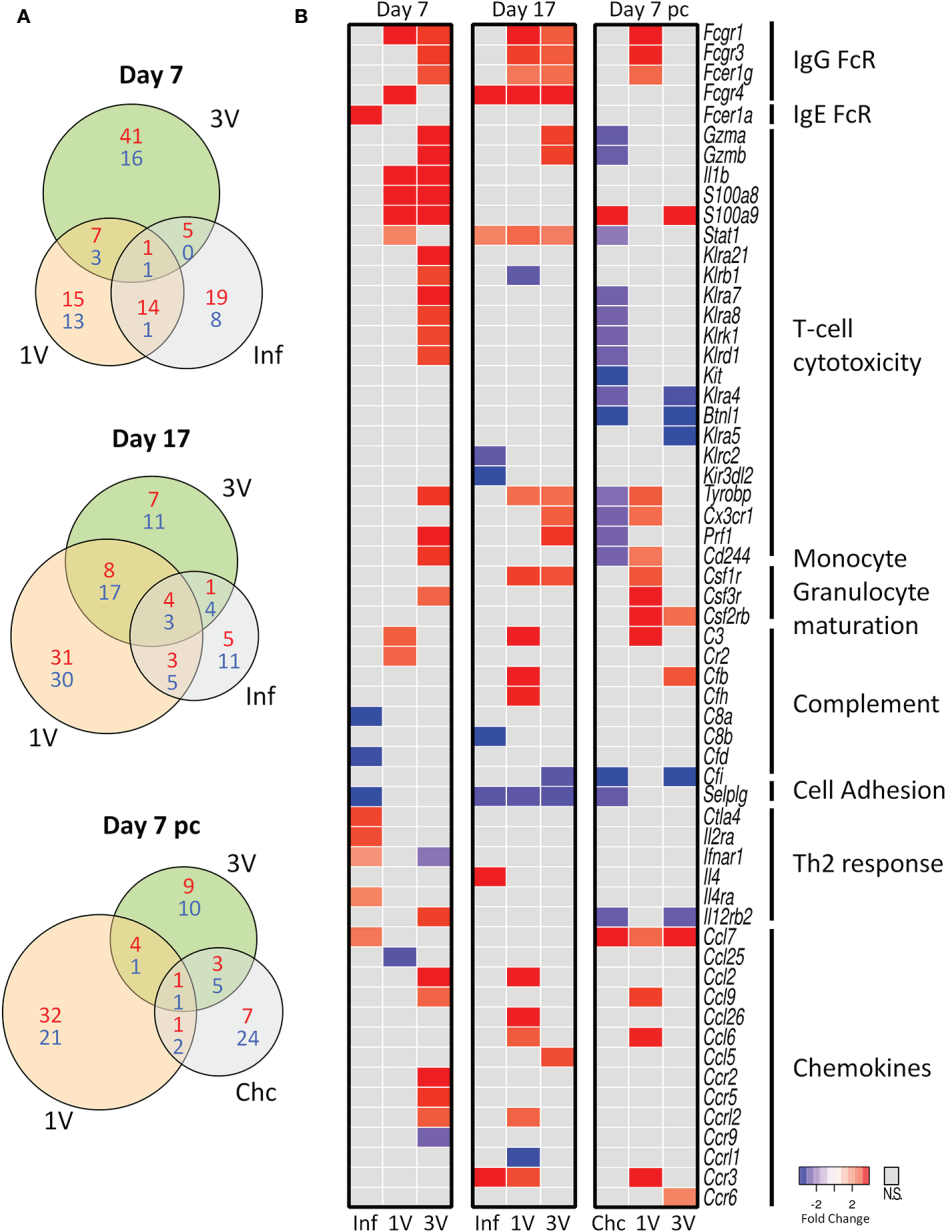
Evaluation of gene expression in PBMC by Nanostring method. **(A)** The shared and unique elements of PBMC by Nanostring analysis showing the DEGs of Inf, 1V and 3V groups at Days 7 and 17 after immunization and Day 7 post-challenge represented in a Venn Diagram (red: up regulated, blue: down regulated) (FDR not adjusted, p < 0.05). **(B)** Heatmaps showing gene expression of a list of 51 differentially expressed genes (DEGs) relative to control PBMC. Data derived from longitudinal assay➀ (nine mice per group). Three biological replicates were assessed per group per time point, in which each individual biological replicate consisted of a pool of PBMCs from three mice (**Supplementary Table 1**).

We analyzed gene expression profiles in PBMCs of immunized and infected animals in the circumscribed Mouse Immunology panel of the Nanostring nCounter^®^ platform; we reasoned that key genes should be revealed between groups at different time points (**Figure 1**). The Venn diagrams revealed a higher number of differentially expressed genes (DEGs) in the 3V group at Day 7 compared to the other groups, whereas DEGs were higher in the 1V group at Day 17 and Day 7 pc (**Figure 4A**). The Inf and Chc groups presented a lower degree of gene perturbation at all time points, suggesting the poor secondary response elicited by normal parasites.

Several genes for Fc receptors, T-cell cytotoxicity, monocyte/granulocyte maturation, complement, chemokine and chemokine receptors were upregulated in the immunized groups at Day 7 and 17 (**Figure 4B**). Conversely, genes involved in Th2 responses were upregulated and some complement proteins downregulated in the infected group; many of the genes involved in T-cell cytotoxicity were downregulated in the Chc group at Day 7 pc (**Figure 4B**). All this amounts to a considerable degree of differential gene expression elicited by the vaccinating parasites, with the 3V group showing the largest number of up-regulated genes. In contrast, the Inf group shows very few up-regulated genes at Day 7, all of which declined by Day 17, thus indicating only a transitory response to the migrating normal schistosomula; the Chc group showed mostly downregulated genes (**Figure 4B**). Of note, are the upregulation of two genes for granzymes (Gzma and Gzmb), alarmins (S100a8 and S100a9) and six killer cell lectin receptors (Klr) in the 3V group before challenge, with many of these downregulated in the Chc group.

At the Day 7 pc sampling time, only the 1V group showed a significant up-regulation of genes, virtually all involved in the arming of monocytes and granulocytes (three Fc receptors, Complement C3, three chemokines and one chemokine receptor), or in the stimulation of their production in the bone marrow (CSF 1, 2 and 3 receptors). One gene, Selplg (P-selectin glycoprotein ligand 1), which plays a crucial role in leucocyte trafficking into tissues, was down-regulated in the infected Inf and Chc groups at all time points analyzed (**Figure 4B**).

Application of the IPA^®^ upstream regulators prediction tool revealed a series of regulators in the 1V and 3V groups and very few in the Inf group. The STAT-1 (transcription activator of interferon-stimulated genes) and PLAU (Plasminogen Activator, Urokinase) were prominent in both vaccinated groups. Activation of interferon regulators (IFN-γ, IFN-α/β and IFN-αr) and inhibition of IL10RA (receptor α chain) appeared in the 1V group. While regulators of IL-12 complex, Interferon alpha, TNFSF12 and SPI1 were active in the 3V group. On the other hand, the ITK and IL-15 regulators involved in T-cell proliferation and cytotoxicity of lymphocytes and NK, respectively, were inhibited in the Chc group (**Supplementary Figure 6**).

### Microarray Analysis of PBMC Expression Reveal Early Activation in Immunization and Down-Regulation of Hemostasis Pathways by Infection

#### Day 7

In order to expand on the results obtained by Nanostring, the whole-genome microarray analysis of PBMC was used to unveil dominant pathways related to the different outcomes observed in immunized and infected animals. Volcano plots illustrate the extent to which gene expression was perturbed in circulating mononuclear leucocytes after exposure to attenuated or normal cercariae. Surprisingly, few DEGs were observed at Day 7 and Day 7 pc, even using less stringent criteria (p<0.001 FDR not adjusted) (**Supplementary Figure 7** and **Supplementary Table 3**). At Day 17 all groups showed considerable changes in gene expression, the top 10 DEGs (up and down) are highlighted in **Supplementary Figure 7B**.

In order to perform comparisons at the different time-points (despite the low perturbation observed at some timepoints), we used GSEA on the entire set of genes detected with microarrays as expressed in PBMC, with the ultimate objective of highlighting new pathways associated with vaccination and infection processes. The identified pathways were manually curated, and those clearly unrelated to immune response or representative of an overly general classification (e.g. DNA replication or cell cycle progression) were excluded from further analysis (all identified pathways are listed in **Supplementary Table 2**).

**Figure 5:**
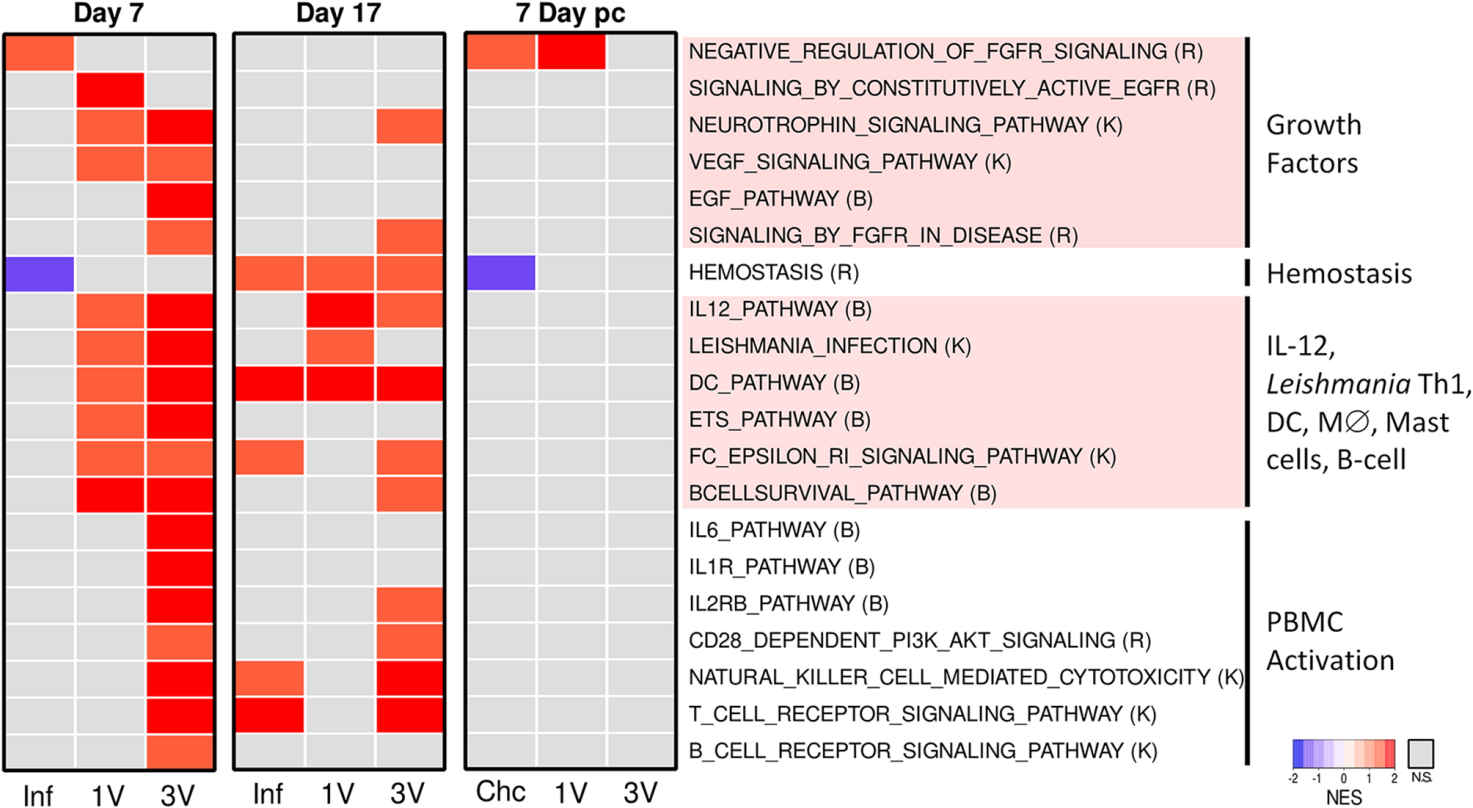
Infection and immunization events induce different gene expression profiles in PBMC. Pathway analysis performed using GSEA (p<0.05) and displayed as heatmaps using the normalized enrichment score (NES) of Inf, 1V and 3V groups at Days 7 and 17 after vaccination/infection and Chc, 1V and 3V groups at Day 7 post-challenge. Data derived from Microarray longitudinal assay➁ with 12–15 mice for experimental groups (Inf, 1V, 3V and Chc), and 22 mice for control group. Four to five biological replicates were assessed per group per time point, in which each individual biological replicate consisted of a pool of PBMCs from three mice (**Supplementary Table 2**). Pathway databases: (R) Reactome, (B) Biocarta, (K) KEGG.

After manual curation and elimination of redundant and overlapping gene sets, a heatmap based on normalized GSEA enrichment scores at Day 7 revealed several pathways that were up-regulated in the 1V and 3V groups compared to the Inf group (**Figure 5**). Notably, multiple pathways related to immune responses were activated in the 1V and 3V groups, including gene sets related to IL-12, *Leishmania* infection, DC, macrophage differentiation, signaling in mast cells and B cell survival (**Figure 5**, **Supplementary Table 2**). In the 3V group, additional pathways observed included IL-6, IL-1R, IL-2RB, NK, and CD28, reflecting a higher level of PBMC activation. In addition, some pathways related to growth factors and their corresponding receptors (NGF, EGF, FGFR, EGFR, and VEGF) were found to be activated in one or both vaccinated groups. In complete contrast, the PBMC signature of the Inf group at Day 7 was minor (**Figure 5**), with the FGFR pathway negatively regulated. Remarkably, the pathway related to hemostasis (activation, signaling and platelet aggregation) was exclusively down-regulated in the Inf group (**Figure 5**).

#### Day 17

At Day 17, the Inf, 1V and 3V groups all showed considerable changes in expression (**Supplementary Figures 7A, B**). GSEA analysis at this time point, the peak of the systemic immune response, revealed less-clear distinctions between the immunized and infected groups (**Figure 5**). However, comparing Day 7 and 17, some changes were apparent, particularly in the Inf group. It is important to remember that by Day 17 normal parasites will have reached the liver, begun to ingest blood and void the gut contents into the bloodstream, whereas attenuated parasites will not have developed beyond the migratory intravascular stage or progressed beyond the lungs. Any distinction or similarity in signature may therefore be due to different causes. In the Inf group at Day 17, multiple pathways were now up-regulated, including hemostasis, plus other pathways associated with T cells and NK cells. In the 1V group, two pathways associated with a Th1 response, namely IL-12 and *Leishmania*, remained up-regulated and distinct from the Inf group. The 3V group remained the most similar to Day 7, but some pathways had diminished, especially growth factors and their receptors but also B cell receptor, IL-6 and IL-1 signaling (**Figure 5**).

#### Day 7 Post Challenge

Analysis of PBMC at Day 7 post-challenge, when the groups had been exposed to 120 normal cercariae (with the Inf group replaced by naïve Chc), revealed remarkably little activity (**Supplementary Figure 7**). GSEA revealed that the challenge controls matched very closely the Inf group at Day 7, with negative regulation of FGFR and the hemostasis pathway (**Figure 5**). The 1V mice showed little evidence for a secondary response to challenge with normal cercariae, with negative regulation of FGFR. The 3V mice, previously primed with three exposures to irradiated parasites were even more refractory, showing only down-regulation of the hemostasis gene set.

### GSEA of sdLN Reveal Signatures for Th1 in 1V, B-Cells in 3V and Hemostasis Pathways in the Inf Group

**Figure 6:**
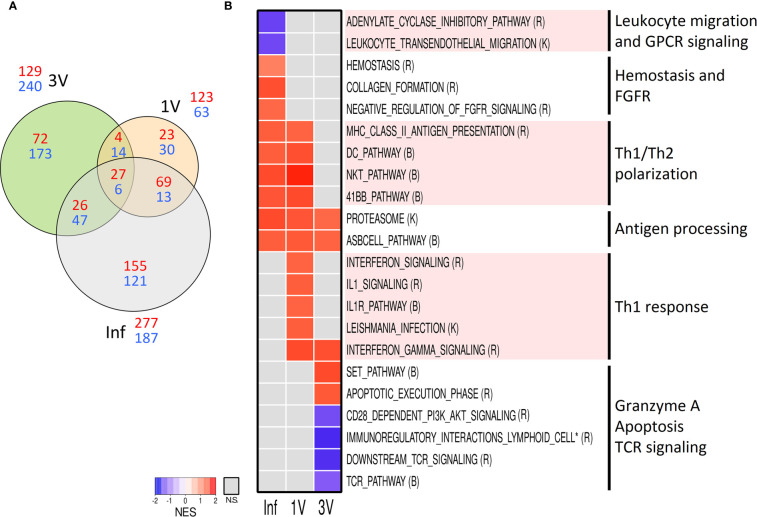
Evaluation of gene expression in sdLN by Microarray analysis. **(A)** The shared and unique elements of sdLN Microarray data showing the DEGs of Inf, 1V and 3V groups at Day 5 represented in a Venn Diagram (red: up regulated, blue: down regulated) (FDR not adjusted, p <0.001); **(B)** Heatmap of GSEA pathways (p <0.05) in sdLN from Inf, 1V and 3V groups at Day 5. Data derived from Cross-Sectional assay ➁ (nine mice per group). Three biological replicates were assessed per group per time point, in which each individual biological replicate consisted of a pool of sdLN RNA from three mice (**Supplementary Table 2**). Pathway databases: (R) Reactome, (B) Biocarta, (K) KEGG.

Aiming to evaluate early signals of immune response in the lymphoid organs, we assessed the gene expression profile in the sdLN at Day 5 after infection or vaccination in an independent cross-sectional assay (**Figure 1**). Volcano plots show the highest number of DEGs in Inf (464), followed by groups 3V (369) and 1V (186) (**Supplementary Figure 8**). The Venn diagram of the DEGs showed that only 33 genes were shared between all three groups, while groups 1V and Inf revealed more similarity (82 shared genes) than the 1V and 3V groups (18 shared genes) (**Figure 6A**).

GSEA was performed on the entire microarray dataset of sdLN in order to reveal early pathways associated with vaccination and infection processes. The pathways were manually curated for the elimination of redundant/overlapping gene sets and those clearly unrelated to immune response or representative of an overly general classification were excluded from further analysis as performed for PBMC (all identified pathways are listed in **Supplementary Table 2**). A heatmap generated revealed distinct signatures in the Inf, 1V and 3V groups (**Figure 6B**). The only gene sets related to immune response shared between all groups was the antigen dependent B-cell activation and the proteasome. Multiple pathways indicative of a Th1 profile were prominent in the 1V group, including gene sets related to signaling of IL-1/IL-1R and signaling related to *Leishmania* infection (which has a Th1 bias), and signaling and regulation of IFN-γ. In addition, several pathways involved in immune activation were shared between the 1V and Inf groups: antigen presentation; dendritic cells; Th1/Th2 polarization; T-cell co-stimulatory signal 4-1BB (CD137). The differentially modulated gene sets exclusive to the Inf group included: hemostasis, collagen formation, and negative regulation of FGFR. The Inf group also showed negatively modulated gene sets involved in leukocyte transendothelial migration and signaling by GPCR. Reflecting the low number of common DEGs, few pathways were shared between the immunized 1V and 3V groups, namely IFN-γ. Among the gene sets confined to the 3V group were granzyme A and apoptosis execution phase. Notably, the 3V group also displayed down-regulated TCR signaling, and co-stimulation by CD28.

## Discussion

Over the last four decades there have been many attempts to develop an effective vaccine for schistosomiasis, but the results have been disappointing. A dissection of the mechanisms occurring in validated models of protection therefore can provide an extra component in the quest for a successful vaccine. Recently, a Systems Analysis approach was applied to the Sm-p80-based vaccines that induce high levels of protection in Baboons (38). Although the study revealed regulation of different pathways, none of them could be correlated with protection levels, precluding identification of a universal signature of immunity.

The live attenuated cercaria vaccine remains the “gold standard” for induction of acquired immunity in rodents and primates, and arguably the best subject for a Systems Analysis of the patterns of gene expression associated with protection. Although the mouse model has limitations, due in part to the low percentage maturation of parasites [reviewed in (39)], its advantage is that the immunological basis of protection has been confirmed, by passive transfer of both antibodies and cells (40–42). There is also extensive knowledge of immune responses, due to the availability of immunological reagents and gene knock-outs (13), as compared to other model hosts such as hamsters and baboons (43).

In our experiments, the mouse model was validated by induction of immune responses and protection, consistent with those established by other groups [reviewed in (3, 10, 12)], achieving >70% reduction in worm burden after three vaccinations. An important effect on female fertility and reduced size of surviving adult worms were also noted. The peak of a T-cell mediated reactivity at Day 17 post-vaccination in the 1V and 3V groups, compared to Inf and Control groups, aligned with the previously described circulating DTH responses in vaccinated animals (31). In addition, the profile of cytokines detected in the plasma and those produced by antigen-stimulated lymphocytes from immunized mice confirmed the production of proinflammatory cytokines, IFN-γ (44) and TNF-α (45), which have well-established roles on the activation of macrophages in the pulmonary effector response (46, 47). A greater recruitment of total leukocytes, macrophages, neutrophils and eosinophils to the lungs was confirmed in 1V and 3V groups, consistent with parasite elimination there (48–50). Higher production of the regulatory cytokine IL-10 after multiple doses of the irradiated vaccine points to an important role in controlling excessive inflammation by down-regulating the development of a highly polarized Th1 response (51, 52).

### How Does the Immune Signature of a Normal Infection Compare With Vaccination?

Differences of gene expression in the sdLN may reflect the reasons why attenuated parasites elicit protection, but normal parasites do not. Initially, there is a marked contrast in parasite migration through the nodes, with around 15% of normal parasites peaking at day 5, but quickly passing through (53) and attenuated parasites being slower to arrive, but persisting there at least to day 15. This persistence brings them into direct contact with antigen-presenting dendritic cells (54), which go on to stimulate strong proliferation of T cells, but not B cells (55). The infected groups displayed early up-regulation of pathways associated with Th2 skewing in antigen presenting cells, revealing a very early predisposition towards a Th2-polarized response, previously thought to depend on the deposition of eggs around week 5; the Th2 polarization is the hallmark of chronic schistosomiasis (56, 57, 58). Our data on the IgG1/IgG2c ratio at Day 28 also support this early polarization towards a Th2 profile, before any egg deposition, and it occurs even in mice with a single sex infection (59).

### Hemostasis

A further distinction is apparent between Inf and vaccinated groups in the regulation of the “pathways of hemostasis”, indicating an important element in the protective immune response. While gene sets for hemostasis (including platelet aggregation) were up-regulated in the Inf and 1V groups at Day 5 in the sdLN, this did not translate to PBMC in the circulation. In the Inf and Chc groups at Day 7 and again at Day 7 post-challenge, respectively, multiple pathways related to hemostasis (activation, signaling and platelet aggregation) were exclusively down-regulated in the PBMCs. We also observed an association between platelet numbers and worm burden, with higher platelet values in vaccinated than infected mice (**Supplementary Figure 9**). Support for this observation comes from one report that *S. mansoni* cercariae depress circulating platelet numbers after infection of mice (60). It has also been proposed that schistosome tegument surface enzymes such as ATPDase 1 can inhibit platelet activation (61). Thus, negative regulation of hemostasis may be a hitherto unsuspected mechanism assisting parasite migration through capillary beds *en route* to the portal vasculature, a process that takes days to weeks. Transit along the tight pulmonary capillaries is a major obstacle (14, 62) and the failure to deploy this evasion mechanism in vaccinated mice could partially explain why their migration is blocked in the lungs (63, 64). It should not be surprising that a migrating schistosome, as a foreign body in intimate contact with the vascular endothelium, would deploy mechanisms to minimize damage that could trigger local hemostasis and impede its progress. Such mechanisms become an obvious target for vaccine-induced protection.

### Leucocyte Migration

The lymphocytes in sdLN and spleen primed by both normal and attenuated parasites are available for recruitment to all tissues, along with inflammatory cells. The current study confirmed recruitment of leucocytes to the lungs of 1V and 3V groups, but not infected mice, as previously determined (49, 65). The schistosome-reactive T cells with a memory/effector phenotype accumulate and arm the lungs against the subsequent arrival of challenge parasites (66), and orchestrate the formation of effector foci to cause their elimination. This element is missing in mice exposed to normal parasites, either because CD4+ T cells with the right characteristics are not generated or because the conditions for large scale pulmonary recruitment are not created.

PBMC reveal a clear dichotomy in chemokine ligand and receptor expression between vaccinated and infected animals. Peak reactivity of 1V animals was observed at Day 17 and again at Day 7 post challenge (5 and 4 genes), while the 3V group peak was earlier at Day 7 (5 genes). In complete contrast, the Inf and Chc mice expressed only 1 or no genes at the three sampling times, revealing their much lower capacity to mount an inflammatory response against the developing parasites. Lung schistosomula are known to modify the functionality of capillary endothelia inducing an anti-inflammatory phenotype (67) and to down-regulate the expression of the adhesion molecules E-Selectin and VCAM-1, induced by TNF-α on vascular endothelia *in vitro* (68). Thus, it appears that the absolute requirement for TNF-α production induced by vaccination (45), overrides an anti-inflammatory mechanism deployed by normal schistosomula in the lungs.

Also relevant to leucocyte migration, is the modulation of Selplg, involved in platelet adhesion to endothelia at sites of inflammation (69). In the Inf and Chc groups it is notable that Selplg is down-regulated at Day 7, limiting early cell recruitment. Paradoxically, at Day 17, there was down-regulation of Selplg in all three mouse groups, indicating a strong inhibitory signal for leucocyte diapedesis into the lungs. Plausibly, this initiates the down-regulation of cell recruitment to the lungs.

### Why Does a Th1 Profile Dominate After a Single Vaccination?

**Figure 7:**
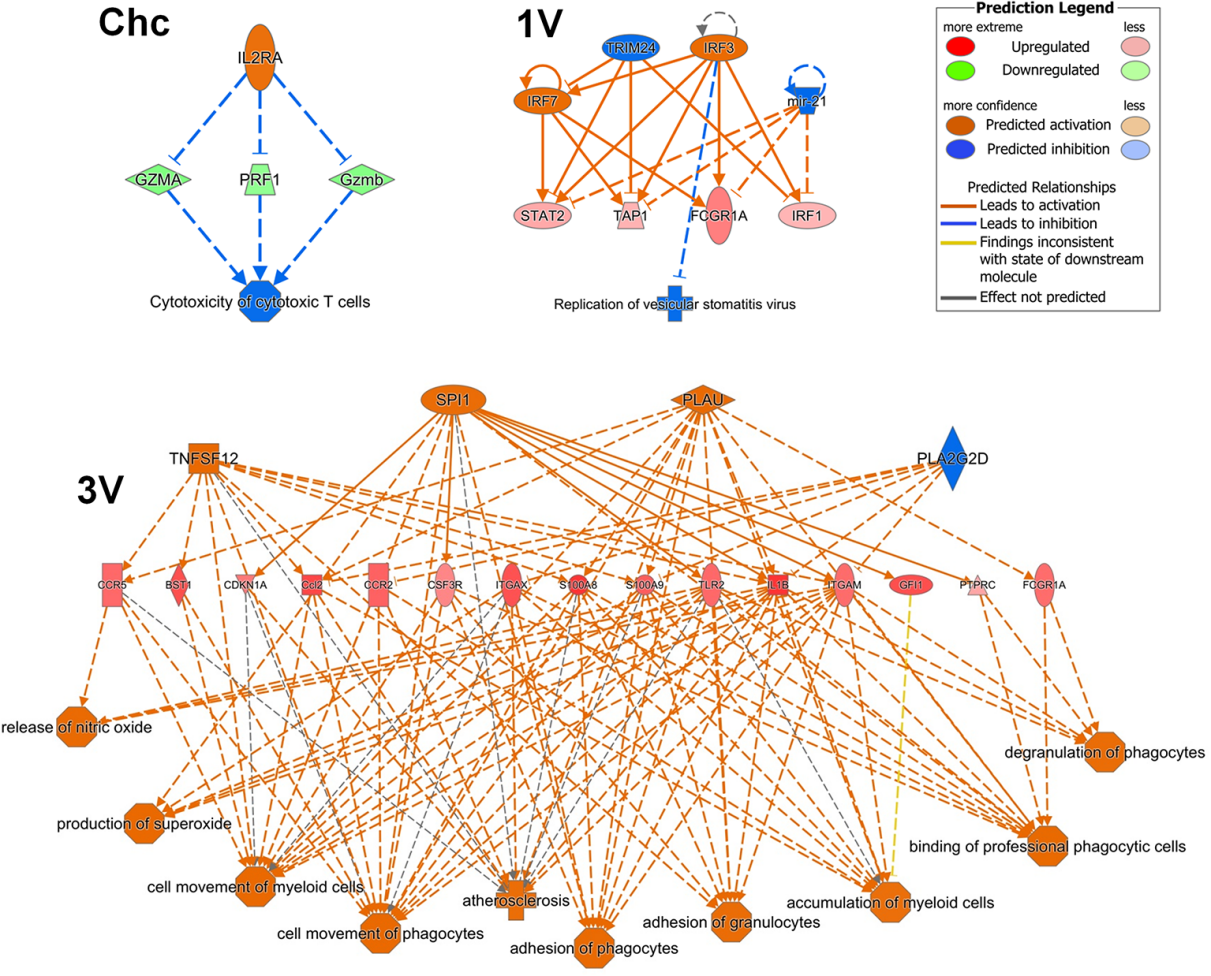
PBMC Regulator Effects Networks analyzed by IPA at Days 7 after vaccination in the 3V group and 1V group and at Day 7 post-challenge in the Chc group. Data derived from longitudinal assay➀.

The detection of a series of Th1 markers by GSEA analysis of the sdLN and PBMC in the 1V group, confirmed the pre-disposition to a Th1 induction pathway as early as five days after exposure to the attenuated parasites, maintained at least until day 17. However, Th2 pathways such as B cell survival and intriguingly, mast cells, were also detected in the PBMC. Finally, IPA upstream regulator analysis of the 1V and 3V Nanostring data supported the induction of the Th1 profile at Day 7 and down-regulation of the IL-10-R alpha chain, removing a potential brake from Th1 expansion. In addition, the regulator effects networks analysis revealed several pathways in the 3V group related to activation and migration of leukocytes (**Figure 7**), considered a sensitive indicator of vaccine immunogenicity (70). On the contrary, the challenged group exhibits negative regulation of cytotoxic T cells (**Figure 7**). We conclude that the extended residence of attenuated, as distinct from normal larvae, in sdLN creates the conditions for prolonged expansion of the Th1 arm in the absence of down-regulatory factors such as IL-10, and the subsequent circulation of these schistosome reactive T cells in the bloodstream.

### The Impact of Multiple Vaccinations and Possible NK Cell Involvement

One of the mysteries of the RA vaccine model in mice is why multiple vaccinations do not dramatically increase the level of protection. The sdLN of the 3V group displayed down-regulation of pathways associated with expansion of T cell populations, such as CD28 costimulation and T cell receptor. Day 7 and 17 revealed the pathway for B cell survival, while the Nanostring analysis revealed up-regulation of IgG-Fc receptors. Our data confirm that after three vaccinations, by Day 28, the IgG1:IgG2c ratio has moved towards the Th2 pole, but not as far as in the Inf group. These observations point to a more active involvement of antibodies in the effector response with increasing vaccinations, correlating with the higher levels of IgG observed in 3V versus 1V animals and lower worm burden.

The GSEA analysis at Day 17 revealed many more active pathways in the 3V than in the 1V group. Given the lack of activity in the sdLN of 3V animals, a possible explanation may be that the 3V circulating PBMC have been mobilized from other sites. Several pathways related to growth factors and their corresponding receptors (NGF, EGF, FGFR, EGFR, and VEGF) were especially enriched in the 3V group. It is unclear what this plethora of growth factors and their receptors might have on vaccine efficacy, but analysis of other vaccines has also revealed several pathways related to cell proliferation, such as EGFR (70, 71).

The Nanostring analysis also highlighted up-regulation of two Alarmin genes (S100A8 and A9) in PBMCs from both 1V and 3V groups at Day 7. Their primary source is neutrophils and they are part of the innate immune response. Alarmins were also observed in immunized baboons (26). A role in protection against schistosomes remains to be established, but their detection in the Chc group at Day 7 suggests a bystander effect from neutrophilia.

Analysis of the sdLN of 3V animals highlighted Granzyme A pathways together with Apoptosis. Additionally, Nanostring analysis of PBMC from the 3V group at Day 7 showed up-regulated expression of GzmA and GzmB and six klr (killer cell lectin) genes, while at Day 17 the two granzymes were still up-regulated. These genes and pathways are all indicators of cellular cytotoxicity. GzmA and GzmB genes encode two Granzyme proteases secreted primarily from cytotoxic (CD8^+^) T cells and NK cells. However, selective ablation of CD8+ T cytotoxic cells in mice immunized once with the RA vaccine revealed they were not essential for protection (72). Similar experiments have not been performed with NK cells, but lymph node NK cells stimulated by IL-12 shortly after vaccination are believed critical to the subsequent development of antigen-specific Th1 cells (73). Recently, a PBMC transcriptomic study in schoolchildren revealed that infection with *S. hematobium* is associated with reduced gene signatures for natural killer (NK) cells, DCs, monocytes and T-cell activation (74). There is also a suggestion from an experiment in pigs immunized with one dose of UV-attenuated *Schistosoma japonicum*, that Granzyme B increase in skin-draining lymph nodes was associated with protection (25).

These observations on NK cell activity and apoptosis in the sdLN of the 3V animals, coupled with down-regulation of T cell costimulatory pathways point to the development of a hyporesponsive state at this site. This would explain why an increase in the number of vaccinations beyond three does not drive the protective response towards sterile immunity. The nodes have been made refractory by down-regulatory mechanisms at the location where priming of T cells occurs. Indeed, multiple exposures of skin to normal schistosome cercariae have been shown to induce hyporesponsiveness in sdLN, implicating IL-10 as the active agent (75).

### Little Evidence for an Anamnestic Response After Challenge

The 1V and 3V mouse groups showed moderate and strong protection, respectively, against establishment of normal challenge parasites, but a striking lack of secondary responses was observed at Day 7 after challenge. Indeed, the genes highlighted by Nanostring analysis in the 1V PBMC were largely directed towards mobilization of antibody effector pathways. Furthermore, the more highly protected 3V group showed minimal perturbation of gene expression after challenge. In earlier studies no recruitment of ^51^Cr-labelled cells to the lungs was observed after percutaneous challenge (65). Additionally, LN draining the site of a challenge in once-vaccinated mice did not generate a secondary Th1 response; instead, cell proliferation and IFN-γ production were profoundly down-regulated (52). Collectively, these observations indicate that the tendency towards a low anamnestic response, already evident after a single vaccination, is so firmly established after three vaccinations as to preclude any further boosting. The persistence of a Th1 signal after three vaccinations (e.g., STAT-1 and IL-12 pathways revealed by IPA analysis of Nanostring PBMC data) may provide an explanation. Experiments using IFNγ R KO mice showed a moderate level of protection after three to five vaccinations (76), even with antibody titers threefold that of vaccinated wild-type animals (13). This observation has clear implications for the type of immune response that might be elicited by vaccination with antigen preparations.

### Which Schistosomula Products Interact With Host Physiology?

Our data indicate that the migration of normal schistosomula is associated with the downregulation of hemostasis that likely facilitates their passage through capillary beds. This mechanism is disabled in vaccinated animals, increasing the difficulty of onward migration. This poses a question about the identity of the parasite products that mediate the process.

Recent analysis of gene transcription in *ex vivo* lung schistosomula confirmed the differential expression of the MEG-3 genes and highlighted five members of the MEG-2 family (77). Coincidentally, these same proteins were secreted by mature eggs embolized in intestinal capillaries (78). It is surely more than coincidence that both the intravascular schistosomulum and egg secrete a similar mixture of MEG-2 and MEG-3 proteins. Indeed, a structural analysis of the MEG-3 proteins suggested they interact with receptors on capillary endothelia to facilitate intravascular migration, and they have been likened to the virulence factors facilitating parasite establishment (79). Collectively, the proteins secreted up to the lung stage at Day 7 provide a novel cohort of antigens that need to be evaluated for protective capacity. Vaccine strategies targeting these proteins may inhibit the down-regulation of hemostasis that facilitates parasite migration through the capillaries, increasing vaccine protection. We have recently used epitope mapping with peptide arrays to investigate the reactivity of 44 *S. mansoni* exposed proteins including the MEG-3 proteins, using sera from the 3V immunized group (28).

Certain limitations need to be borne in mind when interpreting the data from this study. We have not analyzed the lung’s transcriptome (the site of parasite elimination) and the dynamic range of gene alterations in the PBMC and even sdLN was quite low. However, data provided here can guide future experiments using RNAseq or scRNAseq to analyze PBMC, sdLN and lungs after immunization or infection to investigate differences in host and parasite gene expression at high-resolution rate.

Thus, Systems Vaccinology through transcriptomics allowed us to confirm and extend the involvement of genes associated with Th1-immune responses in one-dose and both Th1 and Th2 responses in three-dose vaccination, none of which were activated promptly by infection. Furthermore, it is clear that higher protection was associated with a broader range of activated immune response genes and pathways. The lymph node data revealed early predisposition towards a Th2-polarized response by infection and expansion of Th1 immune responses by vaccination. On the other hand, the PBMC data revealed the regulation of hemostasis pathways as an important mechanism for parasite survival and the role of growth factor signaling in protection, which can help elucidate molecular events responsible for susceptibility and resistance in schistosomiasis, paving the way to immune intervention.

## Data Availability Statement

The data presented in the study are deposited in NCBI's Gene Expression Omnibus (https://www.ncbi.nlm.nih.gov/geo/) repository, series accession number GSE164094.

## Ethics Statement

The animal study was reviewed and approved by the Committee for the Ethical Use of Animals in Experimentation of the Butantan Institute (São Paulo, Brazil) under license 1030/13.

## Author Contributions

LF, JV-S, PH, SV-A, RAW, HN, and LL conceived and designed the experiments. JV-S, PM, DR, MF, RF, TB, GA, and LF performed the experiments and collected data. JV-S, LC, LG, GA, YS, AN, SV-A, RAW, LL, HN, and LF processed and analyzed the data. LF, JV-S, SV-A, RAW, and LL wrote the manuscript. All authors contributed to the article and approved the submitted version.

## Funding

This work was supported by grants from Fundação de Amparo à Pesquisa do Estado de São Paulo to LF and LL (2012/23124-4), Fundação Butantan, and by fellowships from FAPESP to JV-S (2012/18095-5), from Conselho Nacional de Desenvolvimento Científico e Tecnológico (CNPq) to LL, and Coordenação de Aperfeiçoamento de Pessoal de Nível Superior—Brasil (CAPES)—Finance Code 001 to MF. We are grateful to Dr. Pablo I. P. Ramos (CIDACS-IGM-Fiocruz) for his assistance in dataset analysis and Elizabeth S. R. Somessari (IPEN-CNEN/SP-CTR) for her assistance in the cercariae irradiadion procedures.

## Conflict of Interest

The authors declare that the research was conducted in the absence of any commercial or financial relationships that could be construed as a potential conflict of interest.
