# Supplementary material for: Systems Biology Analysis of the Radiation-Attenuated Schistosome Vaccine Reveals a Role for Growth Factors in Protection and Hemostasis Inhibition in Parasite Survival
Source: Front Immunol. 2021 Mar 11;12:624191. doi: 10.3389/fimmu.2021.624191 (PMC7996093; doi:10.3389/fimmu.2021.624191)
Supplement: Supplementary file 1 [file DataSheet_1.pdf]

## Supplementary Materials and Methods

### Blood collection, PBMC separation and RNA extraction

For Microarray and NanoString analysis, 200µL of peripheral blood was collected from the retro-orbital plexus of each mouse and placed in a tube containing 1mL of PBS and heparin (10U, Hepamax-S, 5,000 IU/mL). Diluted blood was then transferred to a 5mL tube and 1mL of Histopaque®1077 solution (Sigma-Aldrich®) was applied slowly via the tube wall to set a gradient. Samples were then centrifuged at 500 x g for 15 min at 20°C. The PBMC ring was removed using a pipette and then transferred to a microcentrifuge tube. Next, two washes were performed with 1 mL of RPMI medium, followed by centrifugation at 250 x g for 10 min under 4°C. Total and viable cells from each animal were counted using an automatic counter (Invitrogen) for assurance of PBMC separation technique quality. Finally, the supernatant was discarded and 200µL of TRizol ® Reagent (Ambion RNA, Life Technologies) was added to the pellet, which was then vigorously vortexed for cell lysis and RNA preservation. All samples were then stored at - 80°C until the time of RNA extraction. Total RNA was extracted using TRizol® Reagent (Ambion RNA, Life Technologies), with a precipitation step incorporating glycogen (5mg/mL, Ambion, USA) plus 3M sodium acetate (Ambion, USA). RNA quality was assessed using an RNA 6000 Nano LabChip Kit (Agilent Technologies), while RNA was quantified using a NanoDrop device (Thermo Scientific). Data revealed a high correlation between the number of PBMCs counted and the amount of RNA extracted (Supplementary Figure 1).

### RNA amplification and labeling

To produce Cy3-labeled cRNA, each pool of total RNA was amplified and labeled using an Agilent Low Input Quick Amplification Labeling Kit in strict accordance with manufacturer instructions. Briefly, 100 ng of template RNA was used for cDNA synthesis, and T7 RNA polymerase was then used for cRNA amplification and Cy3 labeling. After purification, yield and dye incorporation was measured using a Nanodrop spectrophotometer (Thermo Scientific). Sample hybridizations were performed on Agilent slides for 16 h at 65°C. After gentle washing in accordance with Agilent's protocol, microarrays were scanned on a C scanner (Agilent). Agilent Feature Extraction software (v10.7) was used to read and process microarray image files. The software determined feature intensities and background, rejected outliers, and generated validated data files for further analyses. Each array was validated via the analysis of additive error, spatial distribution of outliers in the array, a histogram of signal plots, background sub-signal value and linear range spike-in statistics. All assays were determined to be satisfactory with respect to these parameters.

### Gene Set Enrichment Analysis

The degree of enrichment (enrichment score (ES)) was determined by analyzing gene lists (as present or absent) in a given category, i.e. ES increases when a given gene is present, and decreases in its absence. ES values were normalized (normalized enrichment score (NES)) by adjusting the number of genes in a given category and calculating false detection rates (FDR False Discovery Rate). Gene sets were considered enriched when FDR $\leq 0.05$; this cut-off was used as the main goal of GSEA, as specified by Subramanian and colleagues (37), is to generate hypotheses rather than exclude all false positives. FDR is calculated by comparing the tails of the observed distributions and zero for NES. The null distribution is

produced by assigning random phenotypic labels and producing a list of rearranged genes, performed 1,000 times to generate a null ES for each gene set. We used the gene sets of curated canonical pathways contained in the BioCarta, KEGG and Reactome.

**Ingenuity Pathway Analysis**

We used the commercial QIAGEN's Ingenuity® Pathway Analysis (IPA®, QIAGEN Redwood City) software for upstream regulator and regulator effects networks analysis of identified DEGs. Fisher's exact test was used to calculate the overlap p-value for each analytic tool; significance was attributed to p-values $< 0.05$. For upstream regulators activation or inhibition was considered for Z-scores $\geq 2$ or $\leq 2$, respectively. Theconsistency score of Regulator Effect network means that most of the paths from regulator to target to disease/function are consistent with the predicted state of the regulator based on findings from the literature.
