# Supplementary material for: Systems Biology Analysis of the Radiation-Attenuated Schistosome Vaccine Reveals a Role for Growth Factors in Protection and Hemostasis Inhibition in Parasite Survival
Source: Front Immunol. 2021 Mar 11;12:624191. doi: 10.3389/fimmu.2021.624191 (PMC7996093; doi:10.3389/fimmu.2021.624191)
Supplement: Supplementary file 3 [file Image_2.pdf]

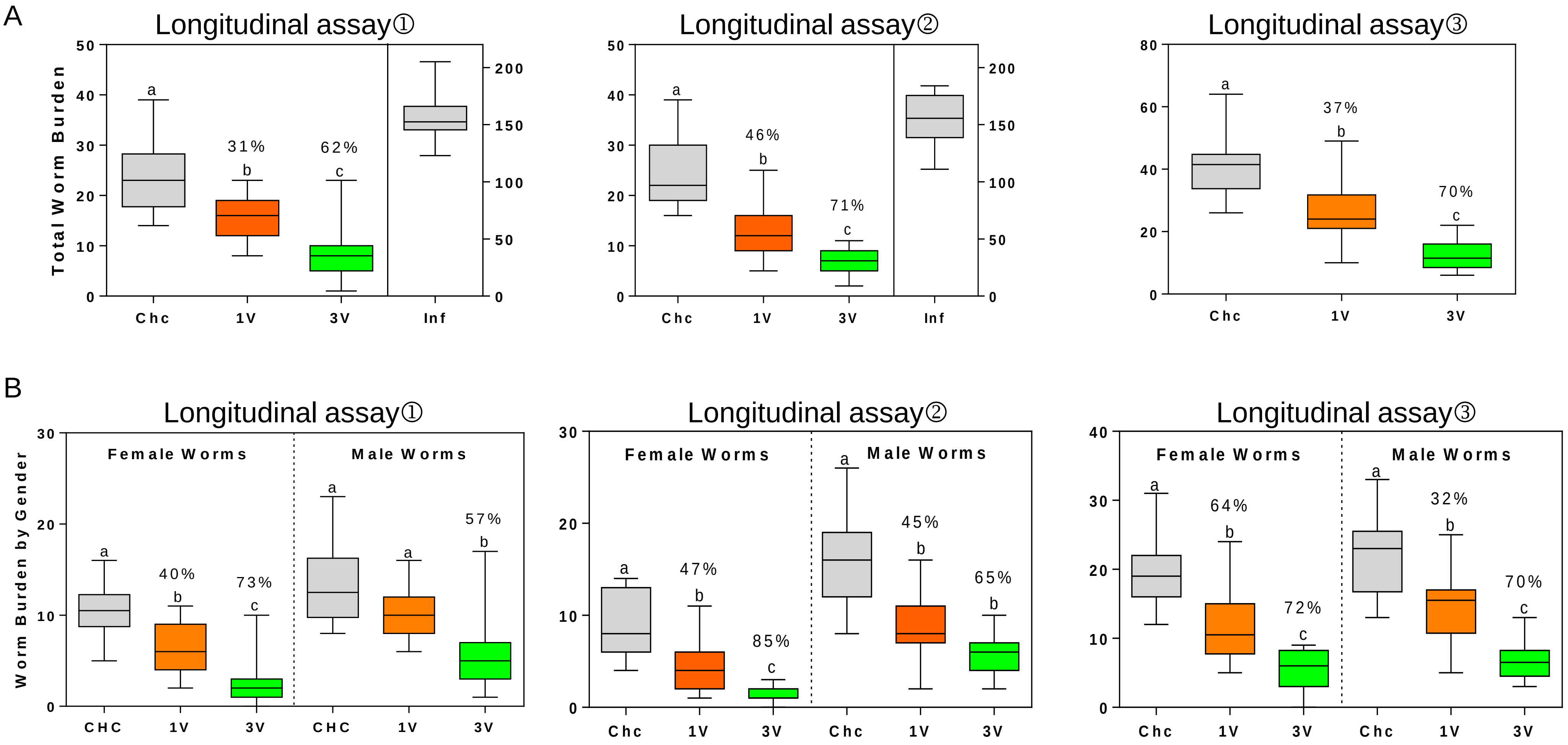

Supplementary Figure 2. Worm burden evaluation from the three longitudinal assays showing the consistency of the RA cercariae vaccine. **(A)** Total worm burden and protection (%) for one-vaccine dose (1V) and three vaccine doses (3V) in comparison to challenge control (Chc). The Inf group was infected with 500 normal cercariae. **(B)** Worm burden separated by schistosome gender (male and female worms) and protection (%) in comparison to Chc. Box plots with different letters represent significant differences; box plots sharing at least one letter represents no significant difference by the Tukey test ($p<0.05$), the Inf group was not included in this analysis. In the longitudinal assay③ worm burden of the Infected group was not recorded. Data was collected from 12-15 mice per experimental group for longitudinal assay① and ② and from 10 mice per group for longitudinal assay③.
