# Supplementary material for: Systems Biology Analysis of the Radiation-Attenuated Schistosome Vaccine Reveals a Role for Growth Factors in Protection and Hemostasis Inhibition in Parasite Survival
Source: Front Immunol. 2021 Mar 11;12:624191. doi: 10.3389/fimmu.2021.624191 (PMC7996093; doi:10.3389/fimmu.2021.624191)
Supplement: Supplementary file 4 [file Image_3.pdf]

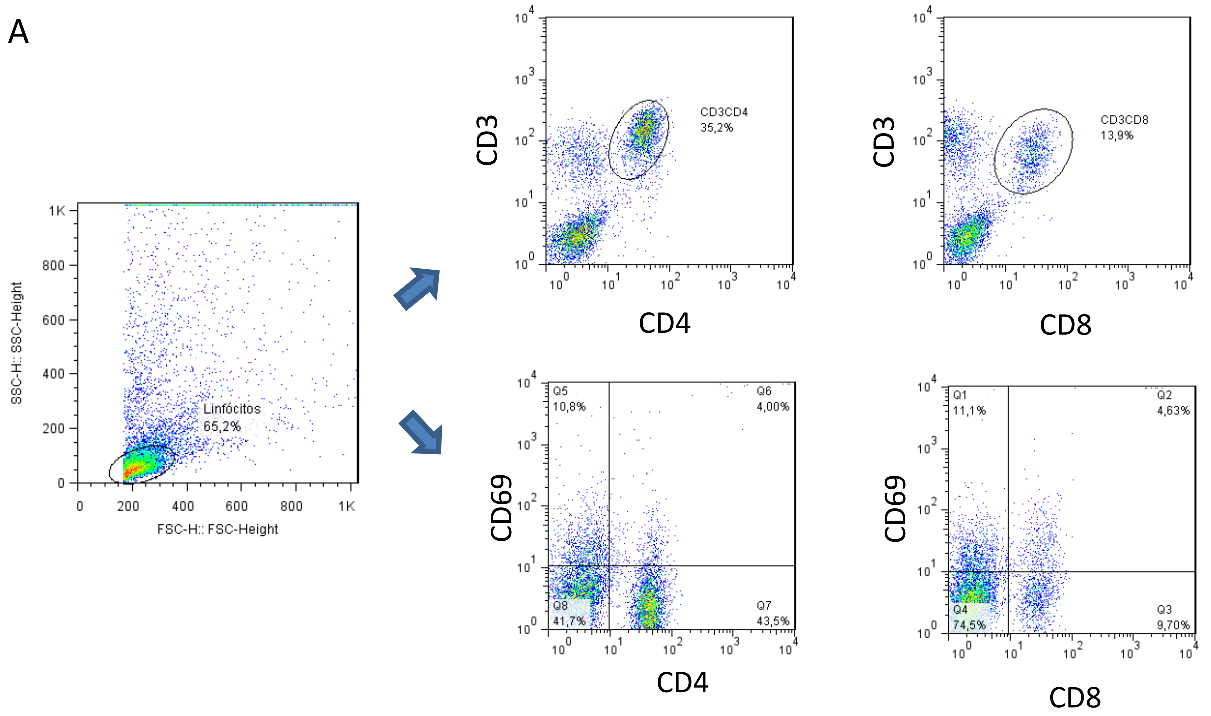

Supplementary Figure 3. Cell recruitment to the lungs and activated lymphocytes in the blood. Effects of immunization (1V or 3V) or infection (Inf or Chc) on the immune response in the lungs and blood at Day 7, 17 and 7 post-challenge. **(A)** Gating strategy for blood cell immunophenotyping presented in (C). For **(B)**, **(C)** and **(D)** see next page.

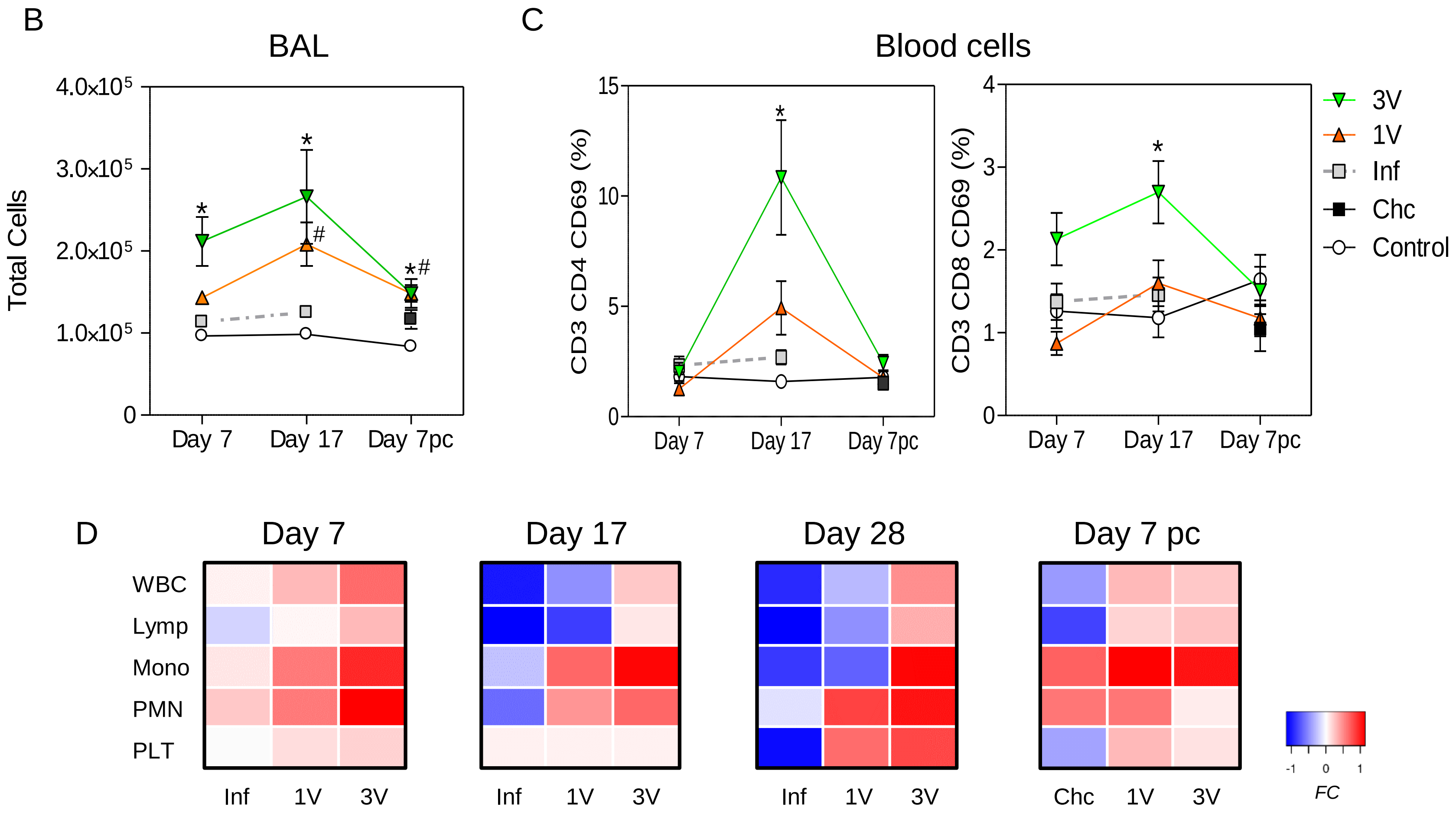

Supplementary Figure 3. Cell recruitment to the lungs and activated lymphocytes in the blood. Effects of immunization (1V or 3V) or infection (Inf or Chc) on the immune response in the lungs and blood at Day 7, 17 and 7 post-challenge. **(B)** Total cells in BAL (bronchoalveolar lavage) fluid. **(C)** Percentage of activated T cells ($CD3^+CD4^+CD69^+$ and $CD3^+CD8^+CD69^+$) in the blood. **(D)** Hematological evaluation, the parameters analyzed were levels of white blood cells (WBC), lymphocytes (Lymp), monocytes (Mono), polymorphonuclear cells (PMN) and platelets (PLT) displayed as heatmaps of the fold change relative to the control group. (*) and (#) indicate statistically significant difference of 3V and 1V in comparison to control group, ANOVA followed by Tukey's post-hoc test (* $p<0.05$). Data for (B) and (C) was derived from cross-sectional assay① (six mice per group per time point); data for (D) was derived from longitudinal assay③ (ten mice per group).
