# Supplementary material for: Systems Biology Analysis of the Radiation-Attenuated Schistosome Vaccine Reveals a Role for Growth Factors in Protection and Hemostasis Inhibition in Parasite Survival
Source: Front Immunol. 2021 Mar 11;12:624191. doi: 10.3389/fimmu.2021.624191 (PMC7996093; doi:10.3389/fimmu.2021.624191)
Supplement: Supplementary file 5 [file Image_4.pdf]

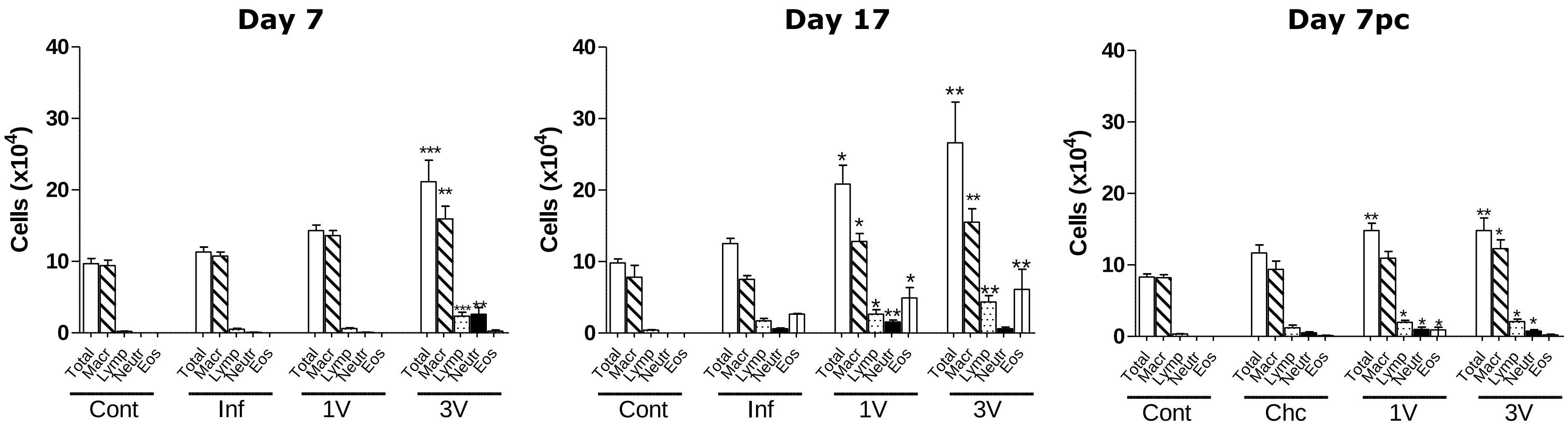

Supplementary Figure 4. Differential count of cells from bronchoalveolar lavage (BAL): lymphocytes, monocytes, neutrophils and eosinophils were evaluated in 1V, 3V, Inf and Chc groups at Days 7, 17 and 7 pc, as appropriate. Asterisks indicate statistically significant difference significance in comparison to control group, ANOVA followed by Tukey's post-hoc test (* $p<0.05$, ** $p<0.01$, *** $p<0.001$). Data derived from longitudinal assay① (6 mice per experimental group per time point).
