# Supplementary material for: Systems Biology Analysis of the Radiation-Attenuated Schistosome Vaccine Reveals a Role for Growth Factors in Protection and Hemostasis Inhibition in Parasite Survival
Source: Front Immunol. 2021 Mar 11;12:624191. doi: 10.3389/fimmu.2021.624191 (PMC7996093; doi:10.3389/fimmu.2021.624191)
Supplement: Supplementary file 6 [file Image_5.pdf]

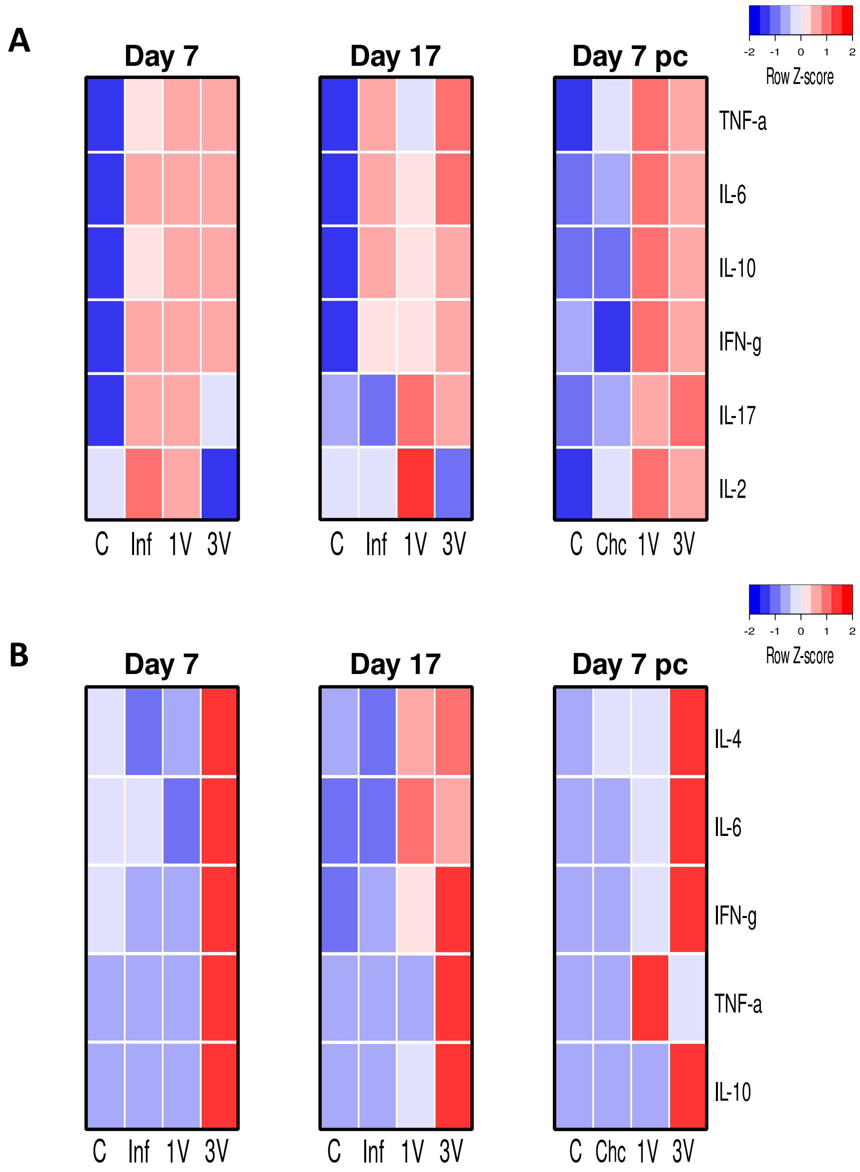

Supplementary Figure 5. Heatmaps depicting Th1/Th2 cytokine production in the spleen culture **(A)** and whole blood **(B)**, colored to indicate levels displayed by Z-score. Control (C), Challenge control (Chc), Infected (Inf), one-vaccine dose (1V) and three vaccine doses (3V) groups at Day 7, 17 and 7 post-challenge. Data was derived from cross-sectional assay① (six mice per group per time point).
