# Supplementary material for: Systems Biology Analysis of the Radiation-Attenuated Schistosome Vaccine Reveals a Role for Growth Factors in Protection and Hemostasis Inhibition in Parasite Survival
Source: Front Immunol. 2021 Mar 11;12:624191. doi: 10.3389/fimmu.2021.624191 (PMC7996093; doi:10.3389/fimmu.2021.624191)
Supplement: Supplementary file 7 [file Image_6.pdf]

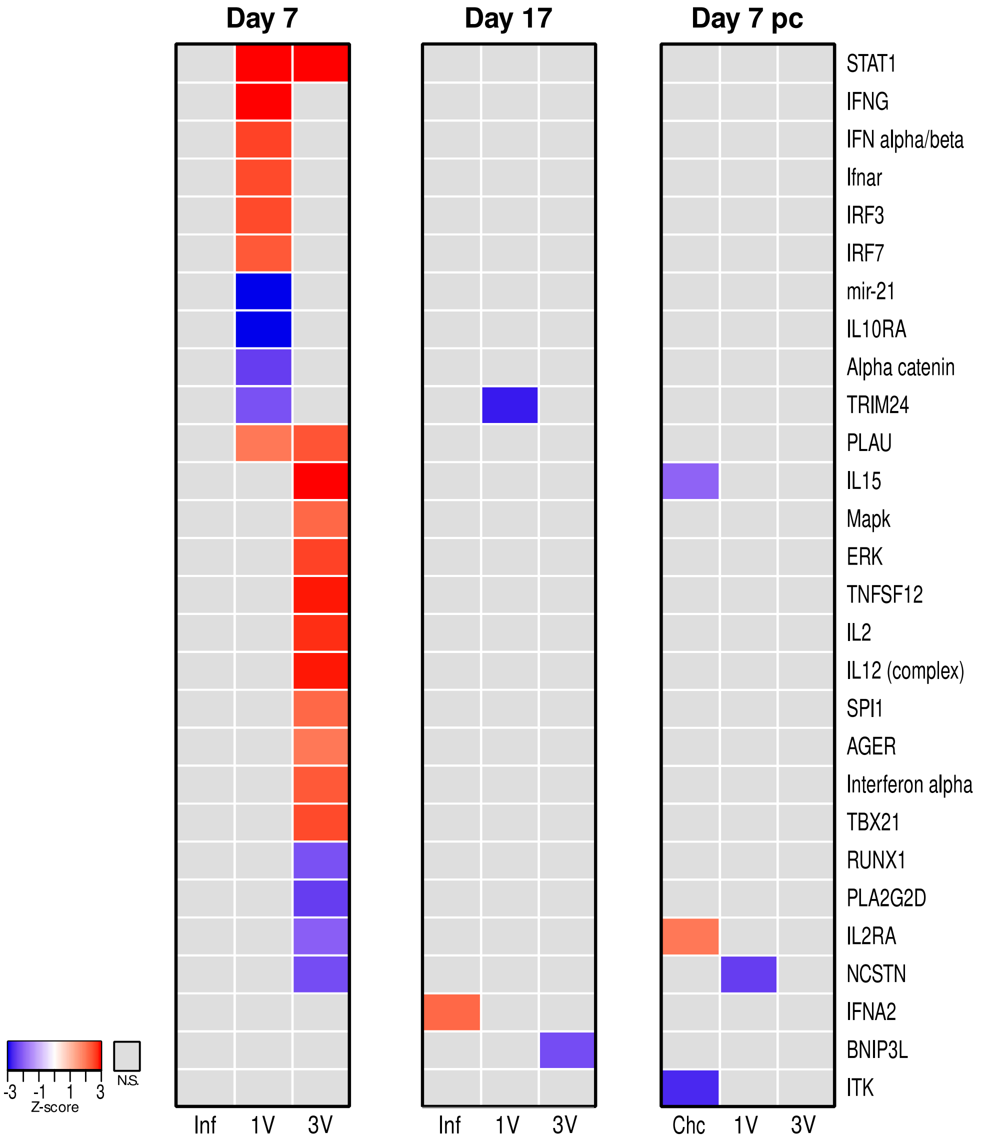

Supplementary Figure 6. Upstream regulators expected to be activated (red) or inhibited (blue) in vaccinated and infected groups. Regulators were identified using the IPA, all regulators represented on the graph are significantly enriched (Z-score ≥ 2 or ≤ −2; *P value* <0.05). Data derived from longitudinal assay①.
