# Supplementary material for: Systems Biology Analysis of the Radiation-Attenuated Schistosome Vaccine Reveals a Role for Growth Factors in Protection and Hemostasis Inhibition in Parasite Survival
Source: Front Immunol. 2021 Mar 11;12:624191. doi: 10.3389/fimmu.2021.624191 (PMC7996093; doi:10.3389/fimmu.2021.624191)
Supplement: Supplementary file 8 [file Image_7.pdf]

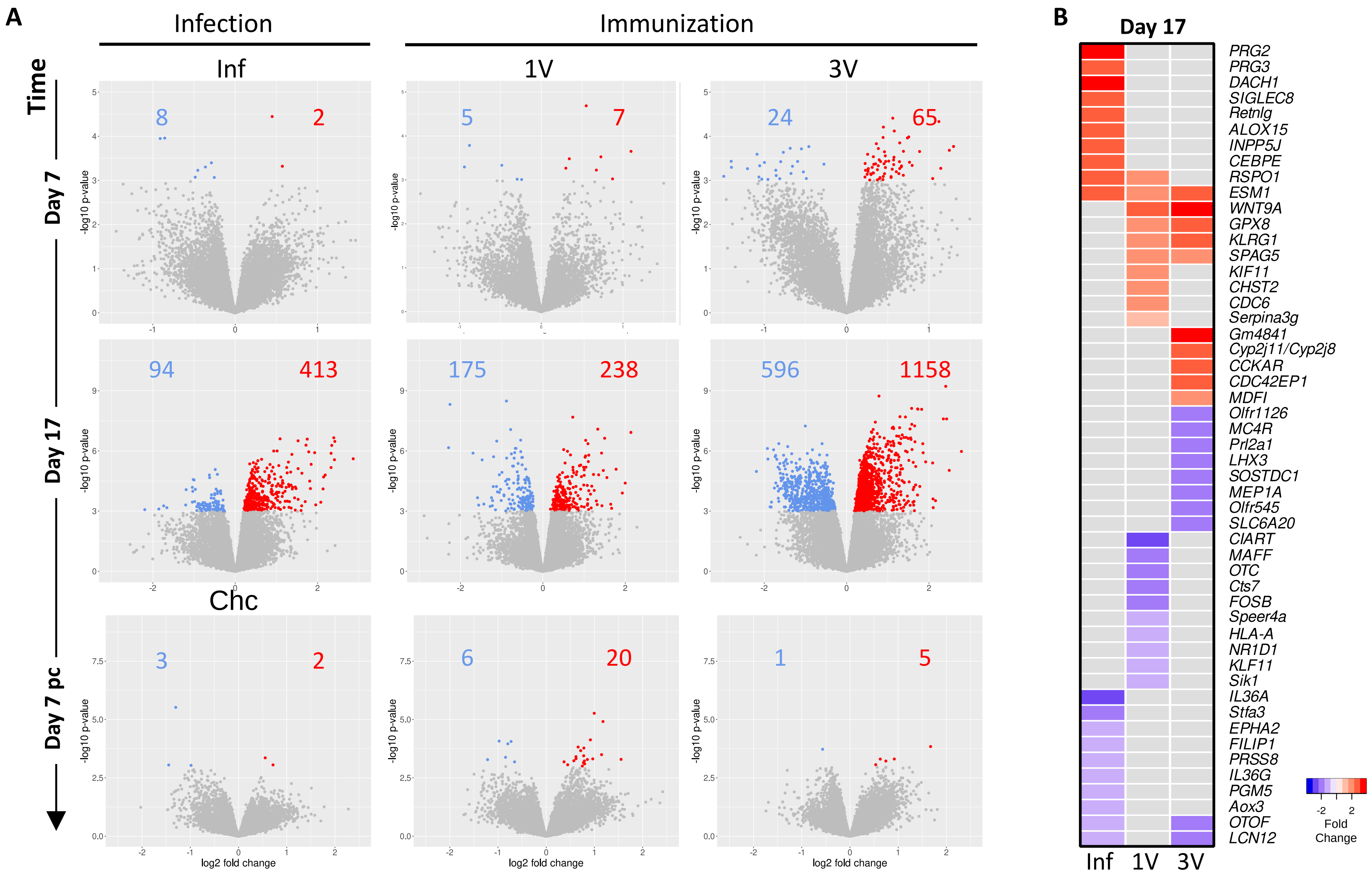

Supplementary Figure 7. (A) Volcano plots from PBMC microarray data showing the DEGs of one-vaccine dose (1V), three vaccine doses (3V), Infected (Inf) and Challenge (Chc) groups as compared to Control at the different time-points (Log(FC) > 0 or < 0, $p < 0.001$, FDR not adjusted). **(B)** Heatmap showing the top 10 DEGs (Up and Down) at Day 17 of Inf, 1V and 3V relative to control PBMC (FDR adjusted, p-value <0.01). Data was derived from longitudinal assay②, in which four to five independent biological replicates (each replicate containing equal amounts of total RNA from three mice, Supplementary Table 2) were assessed per group (Inf, 1V, 3V and Chc) per time point, and 16 biological replicates from control group (C) was used to generate the baseline of gene expression.
