# Supplementary material for: Systems Biology Analysis of the Radiation-Attenuated Schistosome Vaccine Reveals a Role for Growth Factors in Protection and Hemostasis Inhibition in Parasite Survival
Source: Front Immunol. 2021 Mar 11;12:624191. doi: 10.3389/fimmu.2021.624191 (PMC7996093; doi:10.3389/fimmu.2021.624191)
Supplement: Supplementary file 9 [file Image_8.pdf]

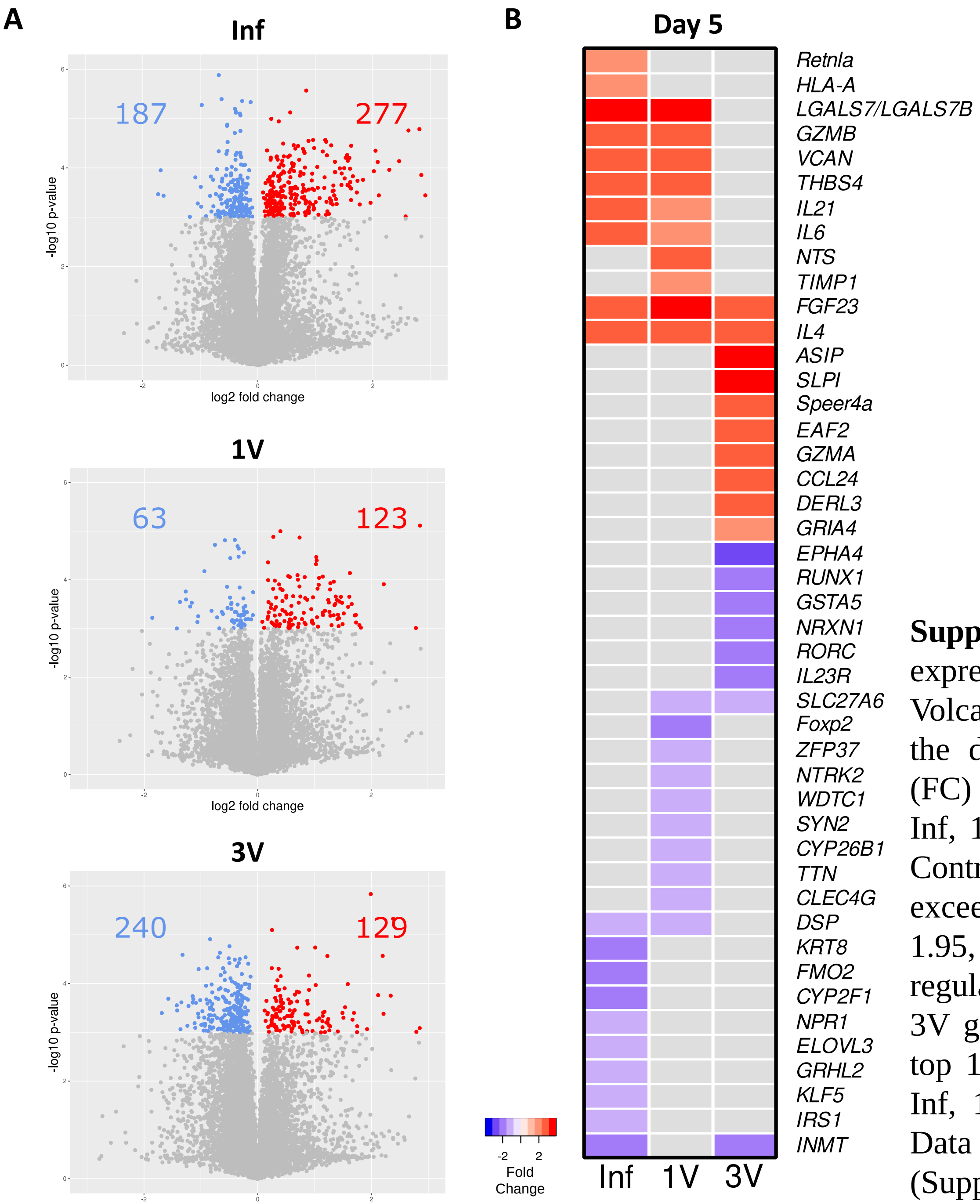

Supplementary Figure 9. Evaluation of gene expression in sdLN by Microarray analysis. (A) Volcano plots of sdLN Microarray data showing the differentially expressed genes (DEGs) (Log (FC) > 0 or < 0, p <0.001, FDR not adjusted) of Inf, 1V and 3V groups at Day 5 as compared to Control. In the Inf and 1V groups, up-regulation exceeded down regulation by ratios of 1.48 and 1.95, respectively, whereas greater down regulation was observed in the multiply exposed 3V group (ratio 0.53). (B) Heatmap showing the top 10 DEGs selected by FC (Up and Down) of Inf, 1V and 3V (FDR adjusted, p-value <0.01). Data was derived from cross-sectional assay② (Supplementary Table 3).
