# Supplementary material for: Systems Biology Analysis of the Radiation-Attenuated Schistosome Vaccine Reveals a Role for Growth Factors in Protection and Hemostasis Inhibition in Parasite Survival
Source: Front Immunol. 2021 Mar 11;12:624191. doi: 10.3389/fimmu.2021.624191 (PMC7996093; doi:10.3389/fimmu.2021.624191)
Supplement: Supplementary file 10 [file Image_9.pdf]

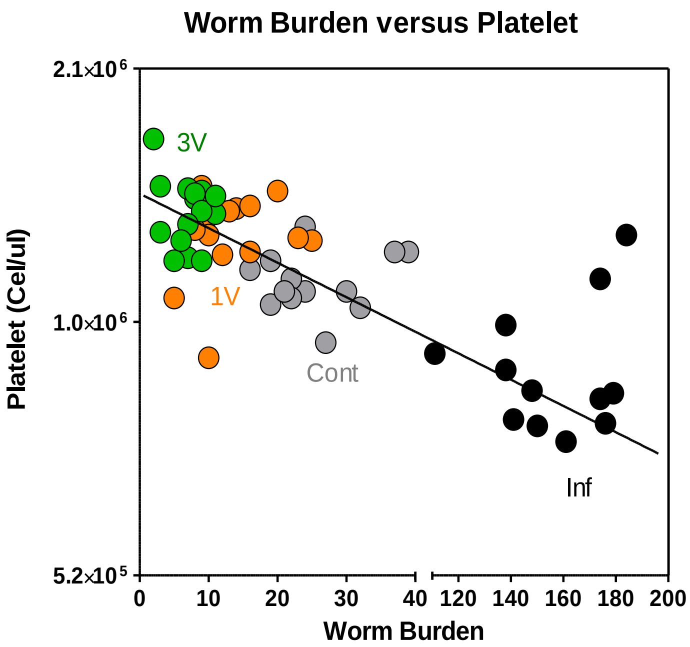

Supplementary Figure 9. Platelets versus Worm Burden. A negative Pearson's correlation between Platelet counts at Day 28 and worm burden at experiment termination was observed analyzing the different groups together ($p<0.0001$: $r = -0.666$).
